# A Novel Inorganic Sulfur Compound Metabolizing *Ferroplasma*-Like Population Is Suggested to Mediate Extracellular Electron Transfer

**DOI:** 10.3389/fmicb.2018.02945

**Published:** 2018-12-05

**Authors:** Gaofeng Ni, Domenico Simone, Daniela Palma, Elias Broman, Xiaofen Wu, Stephanie Turner, Mark Dopson

**Affiliations:** Centre for Ecology and Evolution in Microbial Model Systems (EEMiS), Linnaeus University, Kalmar, Sweden

**Keywords:** bioelectrochemical systems, electricigens, *Ferroplasma*, *Acidithiobacillus*, sulfide mineral mining, metagenome assembled genomes, metatranscriptomics

## Abstract

Mining and processing of metal sulfide ores produces waters containing metals and inorganic sulfur compounds such as tetrathionate and thiosulfate. If released untreated, these sulfur compounds can be oxidized to generate highly acidic wastewaters [termed ‘acid mine drainage (AMD)’] that cause severe environmental pollution. One potential method to remediate mining wastewaters is the maturing biotechnology of ‘microbial fuel cells’ that offers the sustainable removal of acid generating inorganic sulfur compounds alongside producing an electrical current. Microbial fuel cells exploit the ability of bacterial cells to transfer electrons to a mineral as the terminal electron acceptor during anaerobic respiration by replacing the mineral with a solid anode. In consequence, by substituting natural minerals with electrodes, microbial fuel cells also provide an excellent platform to understand environmental microbe–mineral interactions that are fundamental to element cycling. Previously, tetrathionate degradation coupled to the generation of an electrical current has been demonstrated and here we report a metagenomic and metatranscriptomic analysis of the microbial community. Reconstruction of inorganic sulfur compound metabolism suggested the substrate tetrathionate was metabolized by the *Ferroplasma*-like and *Acidithiobacillus*-like populations via multiple pathways. Characterized *Ferroplasma* species do not utilize inorganic sulfur compounds, suggesting a novel *Ferroplasma*-like population had been selected. Oxidation of intermediate sulfide, sulfur, thiosulfate, and adenylyl-sulfate released electrons and the extracellular electron transfer to the anode was suggested to be dominated by candidate soluble electron shuttles produced by the *Ferroplasma*-like population. However, as the soluble electron shuttle compounds also have alternative functions within the cell, it cannot be ruled out that acidophiles use novel, uncharacterized mechanisms to mediate extracellular electron transfer. Several populations within the community were suggested to metabolize intermediate inorganic sulfur compounds by multiple pathways, which highlights the potential for mutualistic or symbiotic relationships. This study provided the genetic base for acidophilic microbial fuel cells utilized for the remediation of inorganic sulfur compounds from AMD.

## Introduction

The extraction of metals from sulfide ores is a robust industrial process carried out in many countries around the world. However, when wastes from sulfide mineral processing are exposed to water and oxygen they can oxidize and form acid mine drainage (AMD), which is characterized by extreme acidity and high metal content (Huang et al., 2016). If released untreated, AMD can damage the environment disastrously and distress the fauna and flora within (Amisah and Cowx, 2000; Hill et al., 2000; Sola et al., 2004). Bacteria and archaea that thrive under acidic environments catalyze AMD formation and these microorganisms are termed acidophiles (pH optimum < 5) and extreme acidophiles (pH optimum < 3). They are often involved in redox transformations of inorganic sulfur compounds (ISCs) and iron containing minerals (Dopson and Johnson, 2012; Johnson et al., 2012) and they have also been exploited for the removal of acid generating ISCs (Liljeqvist et al., 2011). For instance, *Acidithiobacillus caldus* and *A*. *ferrivorans* are known for chemoautotrophic growth on ferrous iron and/or reduced ISCs (Valdes et al., 2009; Hallberg et al., 2010; Mangold et al., 2011; González et al., 2014). *A*. *ferrivorans* is a facultative anaerobe while characterized strains of *A*. *caldus* require oxygen for growth (Hallberg and Lindström, 1994; Hallberg et al., 2010; González et al., 2014). In addition, archaeal populations such as *Ferroplasma* are dominant in extremely acidic AMD environments (Edwards et al., 2000) and published isolates are facultative anaerobes that are capable of chemoorganotrophic growth on yeast extract coupled to the reduction of ferric iron (Dopson et al., 2004).

The emerging biotechnology of microbial fuel cells (MFCs) utilize electrochemically active microorganisms to oxidize substrates and transfer the released electrons to the anode (Logan et al., 2006; Rozendal et al., 2008). From the anode, the electrons flow to the cathode where they are consumed during reduction reactions. This electron flow is harvested as an electrical current and can be used to power low-energy demanding devices such as biological oxygen demand sensors (Di Lorenzo et al., 2014) or to generate useful products such as hydrogen gas (Kuntke et al., 2014). As the MFC technology utilizes microbial catalysis to remediate wastewater, it eliminates the need for the addition of chemical catalysts and recovers electrical energy. Therefore, it is considered energy efficient and renewable (Rozendal et al., 2008). Recently, MFCs have evolved into a versatile technology and their integration with extremophilic microorganisms has widened the choice of electron donors, including inorganic sulfur compounds. As a result, possibilities to treat environmental pollutants under extreme conditions have been opened and MFCs and their uses with extremophiles have been reviewed in Dopson et al. (2016). Recent studies have shown that electrical current (maximum 433 mA m^−2^) can be generated from the degradation of the ISC tetrathionate (S_4_O_6_^2−^) at pH 2.5 (Sulonen et al., 2015) and ISCs in real wastewater from sulfide mineral processing (Ni et al., 2016). These data suggest that acidophile-integrated MFCs are promising for treating AMD-related wastewaters.

The MFC technology is based on the metabolic capability of microorganisms to deliver electrons derived from metabolism across the cell envelope to reduce extracellular electron acceptors, a process termed ‘extracellular electron transfer’ (EET) (Lovley, 2008; Rozendal et al., 2008). This is a widely occurring phenomenon, for instance by dissimilatory metal reducing bacteria such as *Geobacter* and *Shewanella* that reduce metals such as ferric iron or manganese (Nealson et al., 2002; Shi et al., 2007). Such processes are of global importance for iron and sulfur cycling. Currently, molecular characterization of EET mechanisms is limited to *Geobacter*, *Shewanella*, and *Pseudomonas*, revealing three categories including: (i) Outer membrane *c*-type cytochromes for direct electron transfer. For instance, multi-heme *c*-type cytochromes encoded by genes such as *mtrAC*, *cymA*, *omcAESBZ*, and *gspG* in *Geobacter* and *Shewanella* species were shown to be crucial in carrying out EET to metal oxides or MFC electrodes (Holmes et al., 2006; Shi et al., 2007; Bretschger et al., 2008; Richter et al., 2009). (ii) Redox-active molecules (e.g., menaquinone, riboflavin, and phenazines) that function as electron shuttles. It was shown that the *menC* gene is involved in menaquinone and quinone intermediate biosynthesis and its absence severely hampers the ability of *Shewanella* spp. to perform EET (Newman and Kolter, 2000; Myers and Myers, 2004). It was also reported that riboflavin is the key component secreted by *Shewanella* spp. that utilizes the electrode as an electron acceptor (Marsili et al., 2008). Furthermore, phenazines were reported to function as electron shuttles in the reductive dissolution of minerals and the electron transfer to electrode (Hernandez et al., 2004; Wang et al., 2010). (iii) Conductive pili are capable of direct electron transfer, as it was shown that mutants of *Shewanella* deficient in the type II secretion system and type IV pilin are severely limited in the current producing capacity (Bretschger et al., 2008; Richter et al., 2009).

Metagenomics is the cultivation-independent sequencing and characterization of DNA from the microbial community that enables the elucidation of the genetic potential of the entire community (Sharon and Banfield, 2013; Hu et al., 2016). Metatranscriptomics is the profiling of gene transcripts from the complete microbial community and can further elucidate the community’s response to a specific experimental or environmental stimulus. ‘Omics’ studies provide a snapshot of the microbial community profile at the time of extraction. Although the presence of mRNA transcripts does not prove a metabolic process is occurring (Rocca et al., 2015), this approach offers a powerful tool to gain biological insights into the processes of interest. For example, metatranscriptomics can be utilized to identify potential EET stimuli-sensitive genes by comparing gene expression at varying EET rates (Ishii et al., 2013). However, a combined metagenomic and metatranscriptomic investigation of EET has not been conducted on an acidophilic MFC anodic microbiome.

Previously, duplicate MFCs with tetrathionate as substrate and carbon dioxide (as bicarbonate) for carbon source were used to treat ISCs at pH 2.5 (Ni et al., 2016). Tetrathionate was chosen as the substrate as it commonly exists in the flotation process water of sulfide ores (Liljeqvist et al., 2011) and it is stable at low pH. The MFCs were inoculated in the anode chamber with an anoxic sediment from a pH 2.5 – 2.7 AMD stream from the Kristineberg mine, northern Sweden [the AMD conditions are described in Liljeqvist et al. (2015)]. Amplification of the 16S rRNA genes from the anodic microbial consortium aligned within the *Ferroplasmaceae*, *Acidithiobacillus*, *Clostridiaceae*, and *Sulfobacillus* (Ni et al., 2016). Generation of electrical current coupled to tetrathionate degradation was confirmed as being biologically mediated based on (i) cyclic voltammetry scans of the biotic operation showed higher current than the abiotic control; (ii) abiotic operation generated electrical current but it decreased more rapidly compared to biotic operation; and (iii) subsequent inoculation of the abiotic system with the same AMD culture used in the MFCs without additional tetrathionate resulted in an increased cell voltage (Ni et al., 2016).

To unravel the molecular evidence for the degradation of tetrathionate coupled to the generation of an electrical current, we investigated the anodic microbial consortia from previously reported duplicate MFCs (Ni et al., 2016), termed sample ‘S1’ and ‘S2’ for the biological replicate metagenomes and metatranscriptomes. The anode communities’ taxonomic affiliation and metabolic potential were based upon reconstruction of metagenomic-assembled genomes (MAGs). In addition, mRNA transcripts were identified for processes involved in ISC metabolism, EET, inorganic carbon metabolism, and adaptations to conditions within the MFCs. Noticeably, the use of ISCs in the MFC technology under acidophilic conditions is highly novel and molecular EET mechanisms for acidophilic microorganisms are poorly understood.

## Methodology

### DNA, RNA Extraction and High Throughput Sequencing

Planktonic cells were harvested from the anodic compartment of the duplicate MFCs when they were fed with a synthetic media containing 5 mM tetrathionate (Ni et al., 2016). Biomass for DNA extraction was harvested by filtering the anolyte through a 0.2 μm sterile filter (Merck Millipore, United States) when the cell voltage values were 109 and 50 mV from the S1 and S2 MFCs, respectively. Community DNA was immediately extracted using the PowerWater DNA isolation kit (MO BIO, United States) as instructed by the manufacturer. Cells for RNA extraction were taken from the S1 and S2 anodic compartments when cell voltage values were 41 and 53 mV, respectively (Ni et al., 2016). The anolyte was immediately centrifuged at 4°C (10 000 × *g* for 15 min) and the community RNA was extracted according to the manufacturer’s instructions using the RNeasy midi kit for isolation of total RNA from bacteria (Qiagen, Germany). DNase clean-up from the extracted RNA samples was using the Turbo DNA-free Kit (Ambion by Life Technologies, United States) according to the manufacturer’s protocol. The quality and quantity of the nucleic acid samples were tested on Qubit 2.0 (Life Technologies, United States) and Nanodrop (Thermo Scientific, United States). All DNA and RNA samples were sequenced at SciLifeLab, Stockholm, Sweden. The metagenome sequencing was carried out on two Illumina HiSeq 2500 lanes (HiSeq Control Software 2.2.58/RTA 1.17.21.3) with a 2 × 151 bp setup in RapidRun mode. Metatranscriptome sequencing was carried out on a single lane of a HiSeq 2500 (HiSeq Control Software 2.2.58/RTA 1.18.61) with a 2 × 126 bp setup in RapidRun mode without rRNA depletion.

### Bioinformatic Analysis of the Metagenome Data

Bioinformatic analysis of the metagenome sequences (Supplementary File S1) was carried out as described in Wu et al. (2015). Briefly, the raw reads were trimmed, low quality reads and Illumina adapter sequences removed, reads were assembled into contigs using Ray version 2.3.1 (Boisvert et al., 2010) with various k-mer sizes and were combined using Newbler version 2.6. Since ‘binning’ of high throughput sequences into metagenome assembled genomes (MAGs) is a crucial step for the downstream analysis, six strategies (see Supplementary File S2) involving the software CONCOCT version 0.3.0 (Alneberg et al., 2014), MetaBAT (Kang et al., 2015), and MyCC (Lin and Liao, 2016) were assessed by the genome quality software CheckM version 1.0.5. Based upon MAG completeness, evaluation of degree of contamination, strain heterogeneity, and microbial diversity (Supplementary File S2); the best strategy for MAG construction was found to be achieved by individual assembly using CONCOCT with length control of input contigs. Taxonomic information within the MAGs was extracted and phylogenomic trees of the MAGs constructed using PhyloPhlAn version 0.99 (Segata et al., 2013) and visualized using Archaeopteryx (Zmasek, 2012). Functional annotation of each MAG was performed with Prokka (Seemann, 2014) and analyzed using the Kyoto Encyclopedia of Genes and Genomes (KEGG) (Kanehisa and Goto, 2000) and the MetaCyc (Caspi et al., 2014) databases.

### Bioinformatic Analysis of the Metatranscriptome Data

The quality of metatranscriptomic datasets from the anodic microbiomes was checked with FastQC (Andrews, 2010) and trimmed using Trimmomatic version 0.32 (Bolger et al., 2014) with the following parameters: LEADING:20, TRAILING:20, SLIDINGWINDOW:4:25, MINLEN:100. For each sample, the rRNA and mRNA read datasets were extracted with SortMeRNA version 2.1b (Kopylova et al., 2012) with default parameters and rRNA databases provided by the authors on the tool github repository^1^. mRNA reads from the two samples were co-assembled to obtain transcripts using default parameters in Trinity version 2.4.0 (Grabherr et al., 2011). The metatranscriptome assembly quality was assessed with the Trinotate pipeline^2^. Briefly, the representation of full-length reconstructed protein-coding genes was examined by mapping the assembled transcript against the SwissProt database (UniProt Consortium, 2018) using BlastX version 2.6.0+ (Altschul et al., 1990). Read filtering and assembly statistics are reported in Supplementary File S1. The Trinotate pipeline was also used to perform downstream analyses on the assembled transcripts including an estimation of transcript abundance using RSEM version 1.2.29 (Li and Dewey, 2011) and transcript functional annotation using the Gene Ontology (GO) resource (Ashburner et al., 2000). Species activity within the samples was assessed in three complementary procedures: (i) Mapping of mRNA reads to the MAGs with Bowtie2 version 2.2.9 (Langmead and Salzberg, 2012). (ii) Performing a taxonomic assignment of assembled transcripts with Kaiju version 1.5.0 (Menzel et al., 2016) with data reported in Supplementary File S3. (iii) Phylogenetic placement of the rRNA reads. rRNA reads were phylogenetically placed using a reference multiple alignment (RMA) and the related reference phylogenetic tree (RPT) by aligning the reads to the RMA with default parameters in PaPaRa version 2.5^3^ before being inserted into the RPT by re-optimization of RPT edge lengths through the Evolutionary Placement Algorithm (EPA) implemented in RAxML [version 8.2.10; (Stamatakis, 2006)]. RAxML-EPA was used with default parameters except the fraction of insertion branches to be evaluated using slow insertions under ML (-G option) which was set to 0.1, as suggested by the authors. Two RPTs (one archaeal and one bacterial), with the related RMAs and including SSU sequences from Anantharaman et al. (2016) and Hug et al. (2016) were adopted as references to perform the phylogenetic identification. Only phylogenetic placements supported by a likelihood weight ratio of ≥0.90 were retained for further analyses. Abundances of reads associated with each tree node were determined with the guppy v1.1 utility of the pplacer version 1.1.alpha17 package (Matsen et al., 2010) and summarized at the genus level. To investigate if the microbial community contained the sulfite converting APS reductase (encoded by the *apr* gene), it was attempted to amplify it from the community DNA from the two MFCs by PCR using published and specifically designed primers specific for the *aprA* or *aprB* genes (Supplementary File S4).

### Accession Number

The raw metagenome and metatranscriptome sequences are available on the NCBI database with the accession number SRP132763^4^.

## Results and Discussion

### Metagenome Assembled Genomes and RNA Transcripts

The anodic biomass from the previously described MFCs (Ni et al., 2016) were used for DNA and RNA extraction in this study. For the metagenomic sequencing, 142 and 145 million read pairs were obtained for MFCs S1 and S2, respectively, and for the metatranscriptomic sequencing, 97 and 172 million read pairs were obtained for MFCs S1 and S2, respectively (Supplementary File S1). Reconstruction of near complete MAGs by the tested binning strategies and their quality parameters including the level of genome completeness and contamination are detailed in Supplementary File S2. Except for the *Ferroplasma*-like MAG in S1, the level of genome completeness was greater than 94.9% across the accepted MAGs and with less than 3.9% contamination. The transcript annotation and abundance (expressed in transcripts per million, TPMs) in the samples are provided in Supplementary File S3. The *Ferroplasma*-like MAG in S1 had an increased number of duplicated single copy marker genes used for the estimation of MAG quality (Supplementary Files S5, S6). However, it was retained as it was one of the dominating populations based upon mapped reads and RNA transcripts.

**FIGURE 1:**
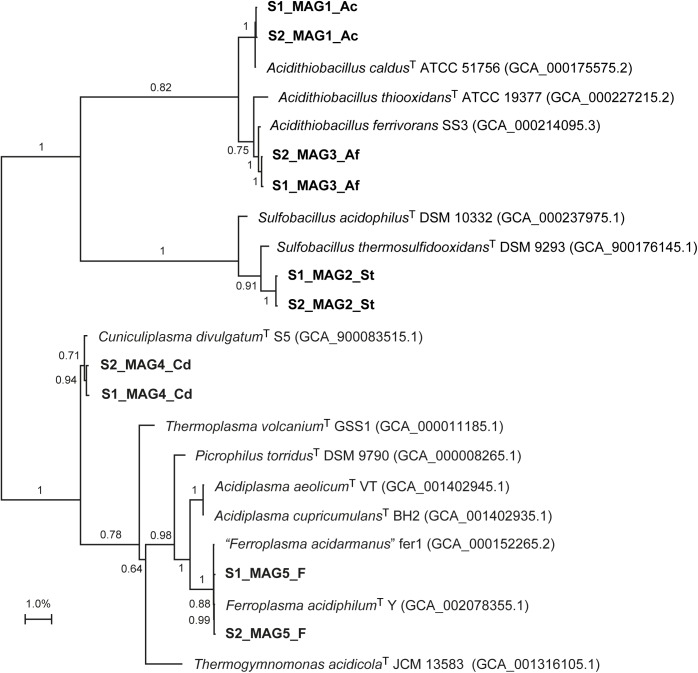
Unrooted maximum distance phylogenomic tree of the MAGs from the duplicate MFCs (in bold) based upon alignment of 400 conserved proteins in the Phylophlan program with confidence values for the nodes (Segata et al., 2013).

Reconstruction of near complete MAGs by the different binning strategies consistently found the dominant populations to be most similar to *Ferroplasma* spp., *A. caldus*, *A. ferrivorans*, *Sulfobacillus thermosulfidooxidans*, and *Cuniculiplasma divulgatum* (Figure 1 and Supplementary File S5). This was in agreement with previous 16S rRNA gene amplicon data that suggested the duplicate anode compartments contained communities that aligned with the *Ferroplasmaceae*, *Acidithiobacillus*, *Clostridiaceae*, *Sulfobacillus* and unclassified species (Ni et al., 2016). Despite the inocula being from different countries, this community was similar to that from another tetrathionate degrading MFC that amongst other populations, also contained *Acidithiobacillus* spp. and “*Ferroplasma acidarmanus*” (Sulonen et al., 2015). Several of the MAGs were most similar to sequenced genomes of cultured species with expected growth characteristics for an ISC metabolizing anode community (Supplementary File S7). For instance, *A. ferrivorans* and *S. thermosulfidooxidans* are facultative anaerobes capable of oxidizing ISCs (Bridge and Johnson, 1998; Hallberg et al., 2010). However, some characteristics of the phylogenetically closest cultured representatives were surprising to be present in an anaerobic anodic community. For instance, *Ferroplasma* spp. have not been demonstrated to oxidize ISCs (Golyshina et al., 2000; Dopson et al., 2004) and *A. caldus* is an obligate aerobe (Hallberg and Lindström, 1994). The fact that the community mediated electrical current generation from the ISCs indicated that potentially novel populations had been selected in the anaerobic anode compartment of the MFC.

**FIGURE 2:**
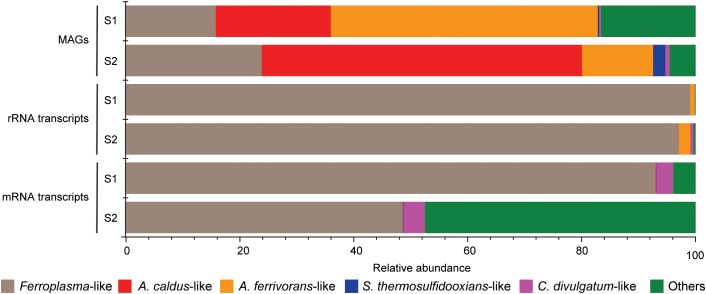
Relative abundance of the mapped reads that were assigned to the MAGs **(top)**, extracted rRNA reads from the metatranscriptome that were assigned to the MAGs **(middle)**, and mRNA reads mapped to the MAGs **(bottom)**.

Mapping of metagenome reads to the MAGs suggested that *Acidithiobacillus*-like populations were the most abundant in the community followed by the *Ferroplasma*-like population, and finally small amounts of *S. thermosulfidooxidans*-like and *C. divulgatum*-like populations (Figure 2). Despite not being most abundant in the community according to the mapping of MAGs, the *Ferroplasma*-like population was the most active at the time of sampling, as suggested by the metatranscriptomic analysis showing that the 99.1 and 97.1% of the 16S rRNA reads were attributed to *Ferroplasma* in samples S1 and S2, respectively, while 93.0 and 48.6% of the mRNA TPMs were attributed to the *Ferroplasmaceae* family in samples S1 and S2, respectively (Figure 2). That the *Ferroplasma*-like population were most active according to the RNA transcripts could have been due to the autotrophic *Acidithiobacillus* and *Sulfobacillus* species being limited by carbon dioxide availability.

### ISC Metabolic Potential Coded Within the MAGs

**FIGURE 3:**
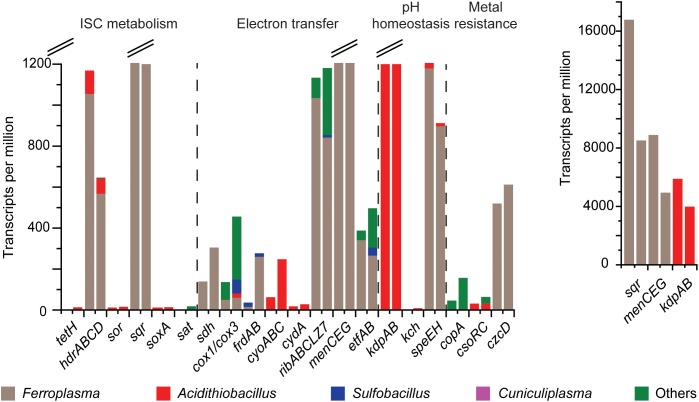
Gene expression profiles for mRNA transcripts annotated (at the genus level) as involved in ISC metabolism, electron transfer to the anode, pH homeostasis, and metal resistance. Genes encode tetrathionate hydrolase (*tetH*), heterodisulfide reductase (*hdr*), sulfur oxygenase reductase (*sor*), sulfide:quinone oxidoreductase (*sqr*), the sox complex (*soxA*), ATP sulfurylase (*sat*), succinate dehydrogenase (*sdh*), cytochrome *c* oxidase (*cox1*/*cox3*), fumarate reductase (*frdAB*), cytochrome *bo*_3_ ubiquinol oxidase (*cyoABC*), cytochrome bd-I ubiquinol oxidase (*cydA*), riboflavin biosynthesis (*ribABCLZ7*), o-succinylbenzoate synthase (*menCEG*), electron transfer flavoprotein (*etfAB*), K^+^-transporting ATPase (*kdpAB*), voltage-gated K^+^ channel (*kch*), spermidine synthase (*speEH*), Cu-exporting P-type ATPase (*copA*), Cu(I) transcriptional repressor (*csoRC*) and Cd/Co/Zn H^+^-K^+^ antiporter (*czcD*). The left-hand bar is for MFC S1 and the right for sample MFC S2. The *y*-axis was split at 1200 TPM and the inset gives total values for *sqr*, *menCEG*, and *kdpAB*.

**FIGURE 4:**
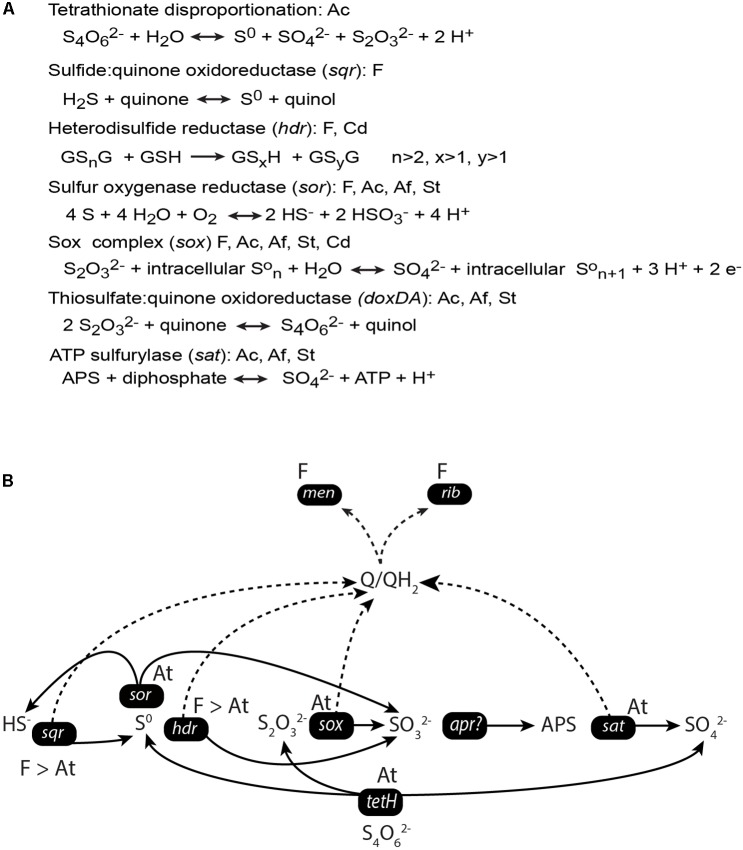
Metabolic potential coded in the MAGs (at the species level) for ISC metabolism in the anode compartments of the duplicate MFCs **(A)** and mRNA transcripts (at the genus level) for the intermediate steps **(B)**. The model **(B)** depicts the genera the genes have been assigned to, the gene names, and the relative number of transcripts assigned to the genera as a greater than sign. The solid line indicates the flow of metabolites, the dotted line represents the flow of electrons. Equations are from MetaCyc (Caspi et al., 2014) except for heterodisulfide reductase (*hdr*) that was adjusted from Rohwerder and Sand (2003). Abbreviations in part **(A)**: F, *Ferroplasma*-like; Cd, *C. divulgatum*-like; Ac, *A. caldus*-like; Af, *A. ferrivorans*-like; and St, *S. thermosulfidooxidans*-like populations. Abbreviations in part **(B)**: F, *Ferroplasma* genus and At, *Acidithiobacillus* genus.

Based on the production of sulfate and elemental sulfur, Sulonen et al. (2015) proposed that tetrathionate was disproportionated in a study with a similar MFC set-up. Due to similarities in the selected microbial communities and the observation of elemental sulfur (a product of tetrathionate disproportionation) on the anode surface, the initial step in tetrathionate metabolism in this study was also likely to be disproportionation (Ni et al., 2016). However, no genetic evidence for this process in an MFC set-up has been presented. Therefore, we investigated for genes and mRNA expression profiles attributed to the metabolism of tetrathionate and other ISCs (Figures 3, 4). The *tetH* gene that codes for tetrathionate hydrolase was detected in the *A. caldus*-like MAGs (Figure 4A and Supplementary File S7), suggesting it was a candidate gene for the initial tetrathionate disproportionation. One product of tetrathionate disproportionation is elemental sulfur that could be metabolized by sulfur oxygenase reductase (encoded by *sor*; present in all populations except the two *C. divulgatum*-like MAGs) to hydrogen sulfide and sulfite (Friedrich et al., 2005). Heterodisulfide reductase (encoded by *hdr*) converts the sulfane-sulfur compound glutathione persulfide (GSSH; a more soluble form of elemental sulfur) to sulfite and glutathione (GSH) under anaerobic conditions (Osorio et al., 2013). The *hdr* gene was present in the *Ferroplasma*-like and *C. divulgatum*-like MAGs, suggesting a second possible pathway for elemental sulfur oxidation. The produced sulfide from sulfur oxygenase reductase (encoded by *sor*) may have been metabolized by the flavocytochrome *c* sulfide dehydrogenase (Chen et al., 1994) encoded by *fccB* present in the *Ferroplasma-*like, *S. thermosulfidooxidans*-like, and *C. divulgatum-*like MAGs or the sulfide:quinone oxidoreductase encoded by *sqr* found in the *Ferroplasma-*like MAGs. Both the gene products of the *sox* cluster and the thiosulfate: quinone oxidoreductase encoded by *doxDA* are predicted to oxidize thiosulfate (Hensen et al., 2006; Mangold et al., 2011; Christel et al., 2016). The genes from the *sox* cluster were present in one *Ferroplasma*-like MAG and all bacteria-like MAGs while *doxDA* was found in the bacteria-like MAGs. Finally, the produced sulfite was suggested to be oxidized to sulfate in two steps of which one is mediated by APS reductase (encoded by *apr*), although this gene was absent in this study and has not been identified in acidophiles [e.g., Quatrini et al. (2009) and Mangold et al. (2011)]. The second step is catalyzed by the *sat* encoded sulfate adenylyltransferase (Quatrini et al., 2009) that was present in all the bacterial MAGs.

The mRNA transcript data (Figure 3) were used to build a model of the community ISC metabolism (Figure 4B). The *tetH* gene for tetrathionate hydrolase had a low number of mRNA transcripts attributed to the *Acidithiobacillus*-like populations (0 and 12 TPM for S1 and S2, respectively). A potential explanation for this was that the community had depleted the available tetrathionate in the anolyte (as supported by chemical measurement, data not shown) and was oxidizing the ISC intermediates (as supported by the high TPMs for *sqr* and *hdr* described below). The starvation of the *Acidithiobacillus*-like population due to limited tetrathionate may explain its relatively lower activity than the *Ferroplasma* population. A second explanation could be that the autotrophic *A. caldus*-like MAGs were limited by the availability of carbon dioxide (detailed in later sections). The majority of the produced elemental sulfur was suggested to be metabolized by heterodisulfide reductase, the product of the *hdr* gene from the *Ferroplasma*-like population (1055 and 568 TPM). An alternative to elemental sulfur degradation was disproportionation catalyzed by sulfur oxygenase reductase (product of *sor*) to form sulfide and sulfite, mRNA transcripts for *sor* was attributed to the *Acidithiobacillus*-like populations (12 and 17 TPM). The produced sulfide may then be oxidized by sulfide:quinone oxidoreductase (product of *sqr*) that had the most abundant transcripts in the metatranscriptome. Based on the mRNA sequence similarity, *sqr* was attributed to the *Ferroplasma* population (16780 and 8509 TPM). Finally, thiosulfate and sulfite were potentially metabolized by *Acidithiobacillus* spp. utilizing the *sox* and *sat* gene products, respectively. mRNA transcripts for *sox* were attributed to *A. caldus*-like MAGs (9 and 14 TPM) while no transcripts were identified for *sat* from the MAGs of this study. The high number of transcripts coding for ISC metabolism enzymes supports the novel finding that a *Ferroplasma-*like population can grow via anaerobic sulfur compound oxidation. Overall, tetrathionate was metabolized by multiple members of the community that suggested the potential for syntrophic or mutualistic interactions between these populations. In addition, the oxidation of sulfide, sulfur, thiosulfate, and sulfite released electrons that likely contributed to the generation of electrical current in the MFCs.

### Electron Transfer to the Anode

Based upon inoculated versus abiotic controls, the previously published MFC study (Ni et al., 2016) strongly supported the occurrence of EET to the anode that generated an electrical current. Consequently, we investigated potential mechanisms for EET based on the metagenomic and metatranscriptomic data. All the MAGs contained a suite of genes attributed to the standard components of respiration including NADH dehydrogenase, succinate dehydrogenase, fumarate reductase, and quinone biosynthesis indicating the presence of electron transfer chains and the ability of the microbial consortium to obtain energy via oxidative phosphorylation (Supplementary File S8). As tetrathionate simultaneously acted as an electron acceptor and donor during disproportionation, the oxidation of the ISC intermediates (e.g., sulfide, sulfur, thiosulfate, and sulfite) released electrons that are transferred to the quinone pool (Dopson and Johnson, 2012). Since electrical current was produced, the microorganisms performed EET on at least a portion of electrons from the oxidation of ISC to the anode as the final electron acceptor.

No genes coding for outer membrane bound multi-heme *c*-type cytochromes were detected in any of the MAGs. Instead, genes encoding the synthesis and secretion of redox shuttles were identified in both the archaeal and bacterial MAGs, including riboflavin biosynthesis proteins and O-succinylbenzoate synthase encoded by *menC* (Supplementary File S8). Although they have other functions within the cell (Wissenbach et al., 1990; Vitreschak et al., 2002), the high number of mRNA transcripts for menaquinone biosynthesis including *menC* (5450 and 3110 TPM for S1 and S2, respectively) followed by *menE* (3232 and 1738 TPM) and *menG* (208 and 102 TPM), as well as for the riboflavin biosynthesis genes *ribABLZ* (1022 and 962 TPM) from the *Ferroplasma*-like population suggested they mediated EET via soluble electron shuttles. Although the known EET mediating *pilA* and *pilD* genes were lacking, other components of the type-IV pili formation genes were identified in all the bacteria-like populations (Supplementary File S8), suggesting that these populations have the genetic potential to conserve energy and carry out EET via conductive pili (Bretschger et al., 2008; Richter et al., 2009). However, mRNA transcripts coding for these genes were not identified. The cultured strain of *C. divulgatum* has the ability to grow anaerobically (Golyshina et al., 2016) and the *C. divulgatum*-like MAGs contained genes annotated for riboflavin biosynthesis proteins expressed at 215 and 345 TPM for S1 and S2, respectively, and O-succinylbenzoate synthase *menC* expressed at 22 and 65 TPM for S1 and S2, respectively. This suggested that the *C. divulgatum*-population might also carry out EET via soluble electron shuttles. As the *C. divulgatum*-like MAGs lack genes for ISC oxidation, it is possible that they grew on organic carbon excreted by acidophiles (Nancucheo and Johnson, 2010) and utilize soluble electron shuttles to carry out EET as an energy conservation strategy. Finally, it cannot be categorically ruled out that a small amount of oxygen leaked into the MFC (such as through the rubber tubing) and this potential alternative electron acceptor could have contributed to the low Coulombic efficiency in the MFCs (Ni et al., 2016). However, aerobic energy conservation was unlikely as seen by lack of mRNA transcripts for *sor*, an enzyme that requires molecular oxygen to oxidize elemental sulfur; as well as the low gene transcripts for cytochrome *c* oxidase for oxygen uptake was limited to just MFC S2 (Supplementary File S3).

Evidence for characterized direct EET via outer membrane *c*-type cytochromes is lacking. Although mRNA transcripts for conductive pili formation were not detected, all the bacteria-like populations contained genes encoding type-IV pili formation, offering genomic capacity for an alternative electrode respiration mechanism. mRNA transcripts supported that the large majority of the EET was potentially mediated by soluble shuttles such as menaquinone and riboflavin produced by members of the *Ferroplasma* genus. Furthermore, it is possible that uncharacterized mechanisms contributed to EET from the acidophiles in this study. It is noteworthy that several known EET mechanisms from neutrophilic populations, e.g., *Shewanella* and *Geobacter* were not detected and this highlights the need to investigate EET mechanisms in acidophiles from both MFCs and natural environments.

### Carbon Fixation Genes Within the MAGs

Mining process and waste waters are typically very low in organic carbon (Nitschke and Bonnefoy, 2016) and the MFC communities were required to fix carbon dioxide for autotrophic growth (Figure 2 and Supplementary File S9). The *Ferroplasma*-like plus the *C. divulgatum*-like MAGs lacked genes for known carbon dioxide fixation pathways and these populations likely grew chemoorganotrophically. This has been shown in a mutualistic interaction whereby autotrophic acidophiles excrete organic carbon that supports chemoorganotrophic growth while chemoorganotrophic populations remove excess organic carbon toxic to autotrophic acidophiles (Slonczewski et al., 2009; Nancucheo and Johnson, 2010). The *Acidithiobacillus*-like MAGs contained genes attributed to the Calvin–Benson–Bassham (CBB) cycle and carboxysome formation while the *S. thermosulfidooxidans*-like MAGs contained *cbb* genes suggesting they are capable of fixing carbon dioxide. The lack of mRNA transcripts encoding carbon fixation (Supplementary File S3) suggested that the carbon dioxide concentration [replenished by carbonate addition in the fed batch MFCs (Ni et al., 2016)] was limiting at the time of cell harvest for metatranscriptomics. This can potentially also explain the lower activity of the *Acidithiobacillus*-like population compared to the *Ferroplasma*-like population as suggested by the RNA transcript abundance data.

### Adaptation to Low-pH Mining Process and Wastewaters

The anodic microbial consortium originated from an AMD environment characterized by low pH and high metal content. To thrive in such environments, acidophilic microorganisms have developed pH homeostasis and metal resistance mechanisms.

Acidophile pH homeostatic mechanisms include membranes resistant to proton influx; an internal positive membrane potential suggested to be generated by potassium ions that inhibits proton influx; the utilization of primary and secondary proton pumps as well as proton consuming reactions; and reduced cell permeability [Supplementary File S10, reviewed in Slonczewski et al. (2009), Zammit and Watkin (2016)]. The need for acidophiles to maintain pH homeostasis in MFCs has a negative effect on cell voltage that lowers power generation and reduced Coulombic efficiency [reviewed in Dopson et al. (2016)]. All of the MAGs contained genes suggested to code for a *kdp* K^+^-transporting ATPase; the *Ferroplasma*-like, the *Acidithiobacillus*-like, and the *C. divulgatum*-like MAGs had a voltage-gated potassium channel *kch* gene; and the *A. caldus*-like MAGs had a low affinity K^+^ *kup* transporter. In contrast, the *Ferroplasma*-like MAG had a pH-gated *kcsA* potassium channel and the *C. divulgatum*-like plus the *Ferroplasma*-like MAGs had a Na^+^/H^+^ antiporter *nhaG* gene. In addition, the bacteria-like MAGs contained one or several Na^+^/H^+^ antiporter genes and all except the *“Ferroplasma*”-like MAGs had an H^+^/Cl^−^ exchange transporter *clcA* gene. Proton consuming reactions include glutamate decarboxylase (Gut et al., 2006) and one or more of the *gadABC* genes were identified in all of the MAGs. Spermidine decreases cell permeability via porins (Samartzidou et al., 2003) and all except one *C. divulgatum*-like MAG had a gene assigned as spermidine synthase (*speE*) while the two *S. thermosulfidooxidans*-like MAGs had the most spermidine/putrescine related genes. The mRNA transcripts suggested that the cells were not under large pH stress as only mRNA transcripts were identified for glutamate decarboxylase and potassium transporters in the *Acidithiobacillus* and *Ferroplasma* genera along with *speE* in *Ferroplasma* (Figure 3).

Mining waters typically contain high metal concentrations requiring the microbial community to be resistant (Dopson and Holmes, 2014) and therefore, the potential metal and metalloid tolerance systems encoded in the MAGs were investigated (Supplementary File S10). All of the reconstructed MAGs contained a minimum of *arsRB* (in the *A. caldus*-like MAGs) to the complete *arsRBCAD* set of genes in the *S. thermosulfidooxidans*-like MAGs suggesting the strains were at least resistant to arsenate (Rosen, 1999). These results were in agreement with characterized strains of “*F. acidarmanus*” (Baker-Austin et al., 2007), *A. caldus* (Dopson et al., 2001), and *S. thermosulfidooxidans* (Guo et al., 2014). All of the bacterial MAGs contained either *copA* or *cus* genes coding for divalent copper and copper/silver resistance, respectively [reviewed in Dopson and Holmes (2014)]. In addition, the *A. caldus*-like and *S. thermosulfidooxidans*-like MAGs contained genes attributed to the *cso* mediated monovalent copper resistance system. The *Acidithiobacillus*-like MAGs had the most gene copies attributed to the cadmium/cobalt/zinc resistance *czc* system. The diverse metal resistance systems in the MAGs are typical of acidophiles inhabiting AMD and reflected the complex multi-metal ore containing zinc, copper, lead, gold, and silver from where the steam flowed from which the inoculum was sampled. That three of the metal resistance related mRNA transcripts were for regulators suggested the cells were not under heavy metal stress in the MFCs containing synthetic media lacking high concentrations of metals (Figure 3).

## Conclusion

Microbe–mineral interactions are fundamental to understand the cycling of major elements on earth and a key process is microbial EET for reduction of oxidized minerals. By replacing natural minerals with electrodes, MFCs are a good platform to understand these processes. Here we report a novel *Ferroplasma*-like population that was suggested to be the most active among the MFC anodic microbial community and metabolized ISCs as well as producing mRNA transcripts encoding redox-active molecules to potentially mediate EET. In addition, the genetic potential and RNA transcript data suggested several populations mediated tetrathionate metabolism. This also suggested symbiotic or mutualistic interactions within the community to carry out multi-species metabolism of ISCs, potential use of soluble electron shuttles, and production of organic carbon by autotrophs that supported heterotrophs. Our findings provided new insights in metabolic capabilities of acidophilic microorganisms and their lifestyle in an engineered environment; elucidated the biological functions of possible future industrial-scale MFCs generating electrical current and remediating mining wastewater as a renewable and energy-efficient approach; and called for future research for EET mechanisms in acidophiles.

## Author Contributions

MD designed the study. GN and ST carried out the laboratory work. GN, DS, DP, EB, and XW carried out bioinformatic analysis and data analysis. GN, DS, ST, and MD drafted the manuscript. All authors commented and approved the manuscript for publication.

## Conflict of Interest Statement

The authors declare that the research was conducted in the absence of any commercial or financial relationships that could be construed as a potential conflict of interest.

## Footnotes

1 https://github.com/biocore/sortmerna

2 https://trinotate.github.io

3 https://sco.h-its.org/exelixis/web/software/papara/index.html

4 https://www.ncbi.nlm.nih.gov/sra/?term=SRP132763
