# Supplementary material for: A Novel Inorganic Sulfur Compound Metabolizing Ferroplasma-Like Population Is Suggested to Mediate Extracellular Electron Transfer
Source: Front Microbiol. 2018 Dec 5;9:2945. doi: 10.3389/fmicb.2018.02945 (PMC6289977; doi:10.3389/fmicb.2018.02945)
Supplement: Supplementary file 1 [file Table_1.docx]

Supplementary Material

A novel inorganic sulfur compound metabolizing Ferroplasma-like population is suggested to mediate extracellular electron transfer via soluble shuttles

**Gaofeng Ni,* Domenico Simone, Daniela Palma, Elias Broman, Xiaofen Wu, Stephanie Turner, and Mark Dopson**

*** Correspondence:**

Gaofeng Ni

Email: Gaofeng.ni@outlook.com

**Supplemental File 1. Sequencing and assembly details for the duplicate metagenomes (top) and metatranscriptomes (bottom) from the MFCs.**

| Sample  ID | Total DNA (μg) | N. of paired reads (M) | N. of trimmed reads (M) | N. of Contigs (> 1000 bp) |
| --- | --- | --- | --- | --- |
| S1 | 3.488 | 142.1 | 276.7 | 2067 |
| S2 | 4.825 | 144.6 | 271.6 | 868 |

| Sample  ID | Total RNA  (μg) | N. of paired reads (M) | N. of trimmed reads (M) | N. of Contigs |
| --- | --- | --- | --- | --- |
| S1 | 3.63 | 96.9 | 48.2 | 6102* |
| S2 | 2.49 | 172 | 96.9 |  |

# *Co-assembly of the two metatranscriptomes

**Supplemental File 2. Evaluation of the near complete MAGs for the six tested binnin strategies.**

| Binning strategy | Bin ID | Completeness (%) | Contamination (%) | Strain heterogeneity (%) | Acceptable | Corresponding MAG |
| --- | --- | --- | --- | --- | --- | --- |
| CONCOCT (cutting up into 10 kbp segments) | 3_S1 | 100.00 | 2.02 | 0.00 | Yes | S1_MAG1 |
|  | 6_ S2 | 99.38 | 2.01 | 0.00 | Yes | S2_MAG1 |
|  | 10_ S2 | 99.34 | 1.24 | 0.00 | Yes | S2_MAG3 |
|  | 9_ S1 | 98.72 | 1.55 | 0.00 | Yes | S1_MAG3 |
|  | 8_ S1 | 98.25 | 124.16 | 97.64 | (Yes)* | S1_MAG5 |
|  | 2_ S2 | 96.33 | 0.87 | 0.00 | Yes | S2_MAG4 |
|  | 8_ S2 | 96.03 | 2.44 | 0.00 | Yes | S2_MAG5 |
|  | 3_ S2 | 96.00 | 3.87 | 0.00 | Yes | S2_MAG2 |
|  | 15_ S1 | 95.52 | 0.00 | 0.00 | Yes | S1_MAG4 |
|  | 4_ S1 | 94.91 | 2.37 | 0.00 | Yes | S1_MAG2 |
|  | 20_ S1 | 92.98 | 7.57 | 94.44 | No | N/A |
|  | 13_ S2 | 80.05 | 17.48 | 92.31 | No | N/A |
|  | 11_ S2 | 8.77 | 0.00 | 0.00 | No | N/A |
| CONCOCT | 2_ S2 | 99.38 | 1.24 | 0.00 | Yes | N/A |
|  | 1_ S1 | 99.38 | 1.24 | 0.00 | Yes | N/A |
|  | 10_ S2 | 99.34 | 1.86 | 0.00 | Yes | N/A |
|  | 7_ S2 | 98.25 | 79.52 | 99.22 | No | N/A |
|  | 12_ S2 | 98.00 | 3.37 | 0.00 | Yes | N/A |
|  | 22_ S1 | 97.30 | 90.95 | 98.12 | No | N/A |
|  | 17_ S1 | 96.33 | 0.00 | 0.00 | Yes | N/A |
|  | 11_ S2 | 96.33 | 0.00 | 0.00 | Yes | N/A |
|  | 13_ S1 | 95.00 | 2.37 | 0.00 | Yes | N/A |
|  | 11_ S1 | 62.50 | 62.50 | 90.00 | No | N/A |
|  | 9_ S1 | 32.14 | 26.16 | 97.83 | No | N/A |
|  | 23_ S1 | 22.47 | 9.79 | 88.46 | No | N/A |
|  | 14_S2 | 10.53 | 0.00 | 0.00 | No | N/A |
| CONCOCT co-assembly of S1 and S2 | 16 | 99.38 | 77.53 | 11.43 | No | N/A |
|  | 8 | 98.00 | 3.37 | 0.00 | Yes | N/A |
|  | 2 | 94.72 | 0.00 | 0.00 | No | N/A |
|  | 14 | 94.13 | 99.93 | 96.57 | No | N/A |
|  | 5 | 45.61 | 1.75 | 0.00 | No | N/A |
|  | 10 | 45.61 | 28.71 | 98.44 | No | N/A |
|  | 17 | 34.35 | 20.42 | 93.48 | No | N/A |
| Metabat  Co-assembly | 1 | 99.38 | 0.00 | 0.00 | Yes | N/A |
|  | 10 | 97.00 | 2.37 | 0.00 | Yes | N/A |
|  | 11 | 96.33 | 0.00 | 0.00 | Yes | N/A |
|  | 4 | 87.88 | 3.73 | 57.14 | No | N/A |
|  | 2 | 82.63 | 29.86 | 90.74 | No | N/A |
|  | 5 | 79.40 | 32.44 | 91.23 | No | N/A |
|  | 3 | 64.91 | 0.00 | 0.00 | No | N/A |
|  | 2 | 10.48 | 0.95 | 100.00 | No | N/A |
| MyCC | 1_S1 | 90.50 | 96.70 | N/A | No | N/A |
|  | 4_S1 | 122.3 | 98.8 | N/A | No | N/A |
|  | 1_S2 | 99.38 | 2.80 | N/A | Yes | N/A |
|  | 2_S2 | 98.00 | 2.37 | N/A | Yes | N/A |
|  | 3_S2 | 99.34 | 12.89 | N/A | Yes | N/A |
|  | 4_S2 | 96.33 | 80.48 | N/A | No | N/A |
|  | 5_S2 | 97.30 | 80.48 | N/A | No | N/A |
| MyCC co-assembly of S1 and S2 | 1 | 99.38 | 2.48 | N/A | Yes | N/A |
|  | 2 | 97.30 | 135.64 | N/A | No | N/A |
|  | 3 | 97.00 | 2.37 | N/A | Yes | N/A |
|  | 4 | 96.33 | 0.00 | N/A | Yes | N/A |
|  | 5 | 66.67 | 67.95 | N/A | No | N/A |

* S1_MAG5 has been included despite having a higher than desired contamination as it was one of the dominating populations based upon mapped reads and RNA transcripts.

**Supplemental File 3.** **Gene name, transcript abundance (TPM), taxonomy ID based on the NCBI database, and lineage (NCBI) for the mRNA transcripts from the S1 and S2 metatranscriptomes.**

A separate Excel file has been uploaded containing this table.

**Supplemental File 4. Supplemental methods and PCR primers used to attempt to amplify the APS reductase coding gene (apr) from the community DNA.**

Nine different published and specially designed primer pairs were tested to amplify the *apr* gene from MFC S1 and S2 community DNA. Primer pairs were designed based on *apr* gene sequences of *Acidithiobacillus sp.* and *Thiobacillus denitrificans* from the Genbank database (accession numbers NVVQ01000014 and AY296750) using Primer3Plus (Untergasser et al., 2007), Beacon Designer^TM^ Free Edition, and Mfold (Zuker, 2003). Specificity of the designed primer pairs was checked via BLASTN 2.8.0 (Altschul et al., 1997). The PCR reactions contained 12.5 µL Phusion High-Fidelity PCR Master Mix (Thermo Fisher Scientific Inc., USA), 0.5 µM of each primer, 2.5 µL BSA solution (3 g L^-1^), 1 µL template DNA, and were filled with distilled H_2_O to the final volume of 25 µL. The PCR conditions were 1 min at 98°C; 35 cycles of 10 sec at 98°C, 30 sec at the respective annealing temperature (see table below), and 20 sec at 72°C; followed by a final extension of 7 min at 72°C. PCR products were analyzed via agarose gel electrophoresis. While all primer pairs resulted in unspecific amplification products of the wrong fragment size (compared to the positive control), primer pairs At_aprFb/At_aprRb, Tb_aprAFb/Tb_aprARb, and Tb_aprBFb/Tb_aprBRb also resulted in products of the correct fragment size. Therefore, for these primer pairs an annealing temperature gradient was tested to improve amplification of the correct-sized products. Amplification with primer pairs Tb_aprAFb/Tb_aprARb and Tb_aprBFb/Tb_aprBRb showed no improvement or unspecific products. However, the amplification for primer pair At_aprFb/At_aprRb resulted in products with the expected fragment size. To check the specificity of these PCR products and the positive control, the bands were cut from the gel, cleaned up with the E.Z.N.A.® Gel Extraction Kit (Omega Bio-tek, USA), re-amplified, and sequenced by Macrogen Inc. (Sanger sequencing; Amsterdam, The Netherlands). The analysis of the sequences revealed that the amplified products of the MFC S1 and S2 were not affiliated to any *apr* gene sequence (BLASTN). In contrast, the positive control was affiliated to *apr* gene sequences of *Thiobacillus thioparus* strain DSM 505, *Thiobacillus denitrificans* ATCC 25259, and *Thiobacillus denitrificans* strain DSM 12475 (query cover = all 99%; identity = 93%, 92%, and 90%, respectively).

Primers and annealing temperature for PCR to amplify the apr gene.

| Primer pair | Primer sequence (5’–3’) | Product size (bp) | T^a^ (°C) | Reference |
| --- | --- | --- | --- | --- |
| AprA-1-FW/  AprA-5-RV | TGGCAGATCATGATYMAYGG  GCGCCAACYGGRCCRTA | 384-396 | 55 | Meyer & Kuever, 2007; Blazejak & Schippers, 2011 |
| aprB2b4_F/  aprB2b4_R | CCNGTNGGCGCNTGGTTC  GGAAGTCTTCCCANGCTTC | 289 | 55 | Lee et al., 2017 |
| aprB3_F/  aprB3_R | GAGAACTTGTGNCCGGA  TGGGAAGABTTCCTVGACATG | 289 | 55 | Lee et al., 2017 |
| At_aprF/  At_aprR | GTGCCAAAGCCGTAATCGTT  TGCCCAATCGTCATACCCAA | 466 | 55 | Designed for this study |
| At_aprFb/  At_aprRb | CGAAGACCTGGGTTACGACA  CGTCCTTGAATCGGGTCAGT | 488 | 55  (48-62)^b^ | Designed for this study |
| Tb_aprAF/  Tb_aprAR | TCCCGAAGTTGTCCAAGAGG  TTGTAGGACTCGCCGTTGAT | 468 | 55 | Designed for this study |
| Tb_aprAFb/  Tb_aprARb | CTGGGCATGGACCTCAAGAT TTGTAGGACTCGCCGTTGAT | 359 | 55  (55-62)^b^ | Designed for this study |
| Tb_aprBF/  Tb_aprBR | GGAGTGCTATTCCTGCGTCA  ACTTGCGAATCAGTTCAGCG | 323 | 55 | Designed for this study |
| Tb_aprBFb/  Tb_aprBRb | GGAGTGCTATTCCTGCGTCA  CTTGCGAATCAGTTCAGCG | 322 | 55  (55-62)^b^ | Designed for this study |

^a^Annealing temperature of the PCR reaction.

^b^Annealing temperature gradient tested to determine optimal temperature.

References:

Altschul *et al.* (1997) "Gapped BLAST and PSI-BLAST: a new generation of protein database search programs". Nucleic Acids Res. 25: 3389-3402.

Blazejak and Schippers (2011) Real-time PCR quantification and diversity analysis of the functional genes *aprA* and *dsrA* of sulfate-reducing prokaryotes in marine sediments of the Peru continental margin and the Black Sea. Front Microbiol 2: 253.

Lee *et al.* (2017). PCR amplification methods for detecting and quantifying sulfate-reducing bacteria in oilfield fluids. Patent Application Publication, Pub. No.: US 2017/0218433 A1

Meyer and Kuever (2007) Phylogeny of the alpha and beta subunits of the dissimilatory adenosine-5’-phosphosulfate (APS) reductase from sulfate-reducing prokaryotes – origin and evolution of the dissimilatory sulfate-reduction pathway. Microbiology 153: 2026–2044.

Untergasser *et al.* (2007) Primer3Plus, an enhanced web interface to Primer3. Nucleic Acids Res 35: W71-W74; doi:10.1093/nar/gkm306

Zuker (2003) Mfold web server for nucleic acid folding and hybridization prediction. Nucleic Acids Res 31: 3406-3415

**Supplemental File 5. Sequence information for each approved MAG.**

| Bins | Estimated genome size (bp) | Genome size of most closely related strains (bp) | No. contigs | n50^a^ | GC% | No. single copy genes^b^ | No. duplicated single copy genes | Contig length cutoff | Mapped reads (%)^c^ | Suggested phylogeny (NCBI) |
| --- | --- | --- | --- | --- | --- | --- | --- | --- | --- | --- |
| S1_MAG1_Ac | 2,970,418 | 2,987,045 | 286 | 10000 | 61.38 | 36 | 0 | 10000 | 20.19 | *A. caldus* |
| S2_MAG1_Ac | 2,926,710 | 2,987,045 | 280 | 10000 | 61.39 | 35 | 0 | 10000 | 56.17 | *A. caldus* |
| S1_MAG2_St | 4,454,149 | 3,861,015 | 482 | 10000 | 57.51 | 33 | 0 | 10000 | 0.24 | *S. thermosulfidooxidans* |
| S2_MAG2_St | 4,609,008 | 3,861,015 | 448 | 10000 | 57.52 | 35 | 0 | 10000 | 2.15 | *S. thermosulfidooxidans* |
| S1_MAG3_Af | 2,992,026 | 3,207,552 | 344 | 10000 | 56.74 | 36 | 0 | 10000 | 46.83 | *A. ferrivorans* |
| S2_MAG3_Af | 3,002,809 | 3,207,552 | 299 | 10000 | 56.83 | 36 | 0 | 10000 | 12.50 | *A. ferrivorans* |
| S1_MAG4_Cd | 1,920,878 | 1,938,699 | 187 | 10000 | 39.32 | 34 | 0 | 10000 | 0.30 | *C. divulgatum* |
| S2_MAG4_Cd | 1,940,791 | 1,938,699 | 191 | 10000 | 39.38 | 35 | 0 | 10000 | 0.71 | *C. divulgatum* |
| S1_MAG5_F^d^ | 5,245,894 | 1,826,943 | 207 | 10000 | 36.60 | 35 | 17 | 10000 | 15.82 | *Ferroplasma* spp*.* |
| S2_MAG5_F | 1,706,562 | 1,826,943 | 164 | 10000 | 36.95 | 35 | 0 | 10000 | 23.88 | *Ferroplasma* spp. |

^a^n50: number of the largest contigs that sum up to 50% of the total sum of bases

^b^Number of single copy genes used in the CONCOCT pipeline.

^c^Mapped reads (%) from respective metagenomes.

^d^ S1_MAG5 has been included despite having a higher than desired genome size as it was one of the dominating populations based upon mapped reads and RNA transcripts

**Supplemental File 6. The results of the CONCOCT binning showing the number of each single copy gene for each sequencing. The color coding shows the number of copies (from zero to four) of the 36 CONCOCTS single copy genes identified in each of the MAGs. Only bins designated as acceptable have been named.**

**S1 bins.**


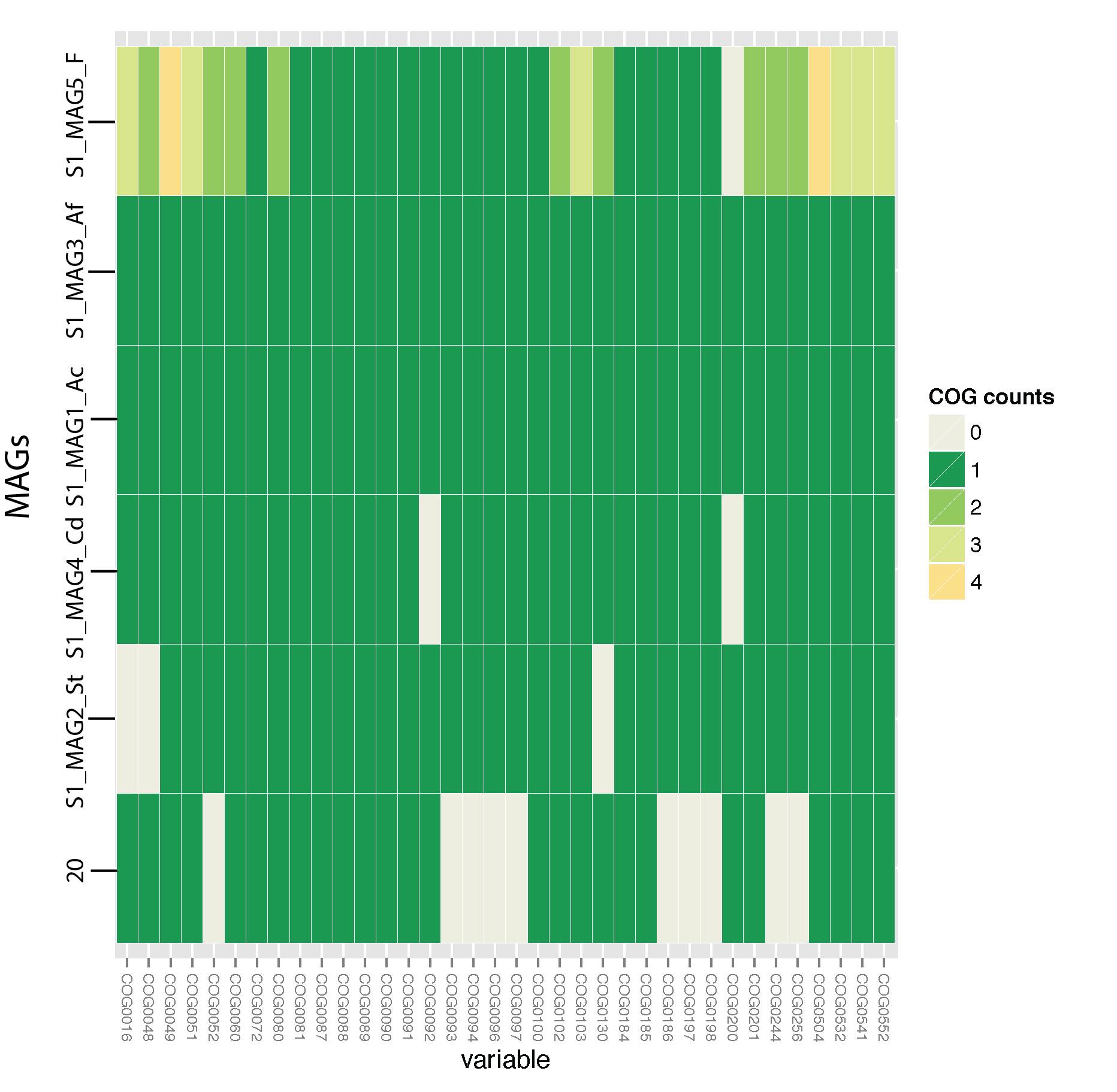


**S2 bins.**


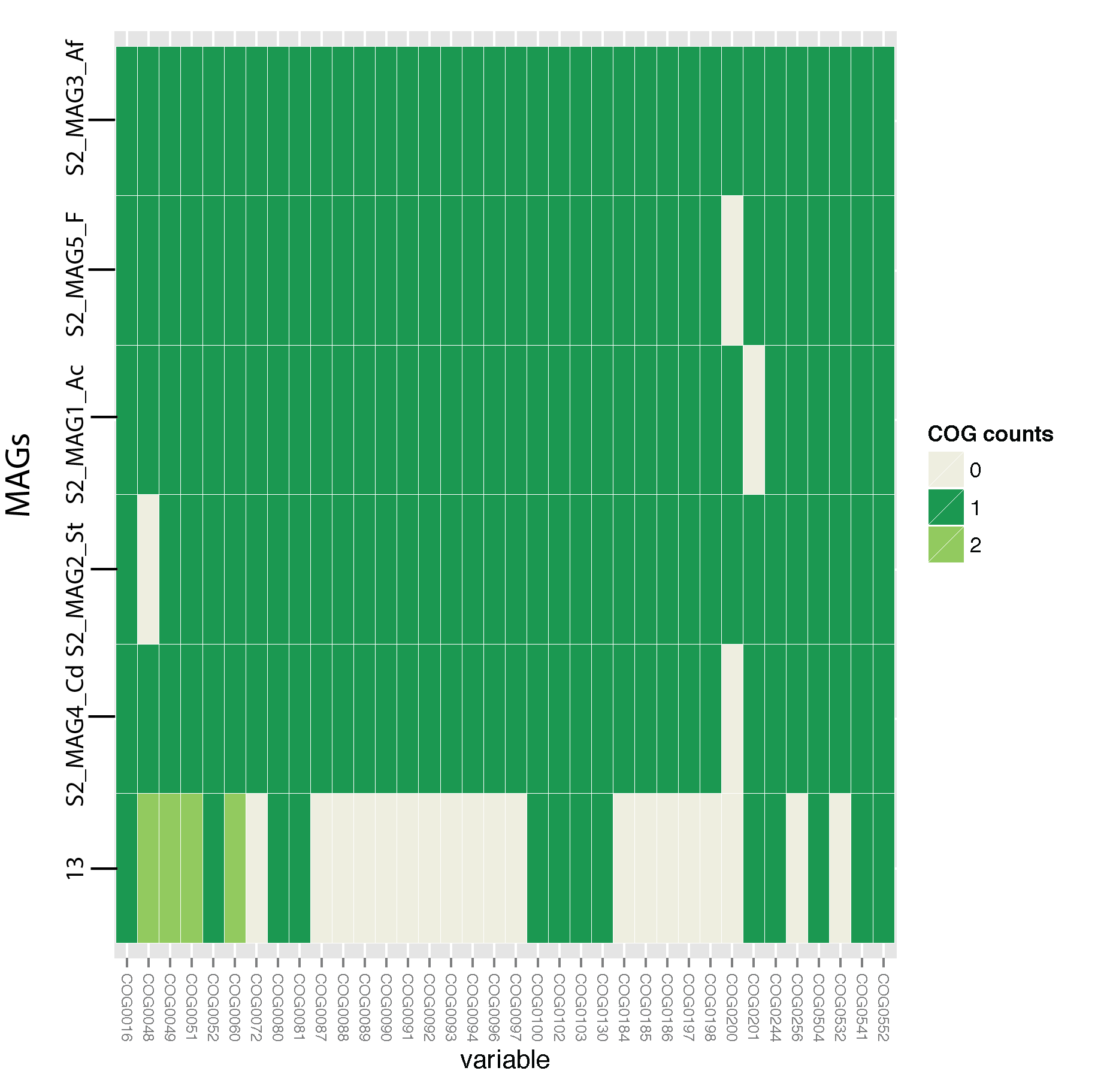


**Supplemental File 7. ISC metabolic potential coded within the MAGs.**

|  | **Number of gene copies identified in the MAGs ^1^** | | | | | | | | | |
| --- | --- | --- | --- | --- | --- | --- | --- | --- | --- | --- |
| **Gene** | **S1_MAG1_Ac** | **S2_MAG1_Ac** | **S1_MAG2_St** | **S2_MAG2_St** | **S1_MAG3_Af** | **S2_MAG3_Af** | **S1_MAG4_Cd** | **S2_MAG4_Cd** | **S1_MAG5_F** | **S2_MAG5_F** |
| **Tetrathionate** |  | | | | | | | | | |
| Tetrathionate hydrolase *tetH* | 1 | 1 | 0 | 0 | 0 | 0 | 0 | 0 | 0 | 0 |
| **Thiosulfate** |  |  |  |  |  |  |  |  |  |  |
| *doxD* | 1 | 1 | 1 | 1 | 1 | 0 | 0 | 0 | 0 | 0 |
| *doxA* | 0 | 0 | 1 | 1 | 0 | 0 | 0 | 0 | 0 | 0 |
| Thiosulfate sulfurtransferase *glpE* | 2 | 2 | 5 | 5 | 3 | 3 | 3 | 3 | 0 | 2 |
| *soxX* | 1 | 1 | 0 | 0 | 0 | 0 | 0 | 0 | 0 | 0 |
| *soxY* | 2 | 2 | 0 | 0 | 2 | 2 | 0 | 0 | 0 | 0 |
| *soxZ* | 2 | 2 | 0 | 0 | 2 | 2 | 0 | 0 | 0 | 0 |
| *soxA* | 2 | 2 | 0 | 0 | 1 | 1 | 1 | 1 | 0 | 1 |
| *soxE* | 0 | 0 | 4 | 4 | 0 | 0 | 0 | 0 | 0 | 1 |
|  |  |  |  |  |  |  |  |  |  |  |
| **Sulfur** |  |  |  |  |  |  |  |  |  |  |
| Sulfur oxygenase reductase *sor* | 2 | 2 | 1 | 1 | 1 | 2 | 0 | 0 | 3 | 1 |
| Heterodisulfide reductase *hdrB* | 0 | 0 | 0 | 0 | 0 | 0 | 0 | 0 | 4 | 1 |
| Heterodisulfide reductase *hdrD* | 0 | 0 | 0 | 0 | 0 | 0 | 2 | 2 | 3 | 1 |
|  |  |  |  |  |  |  |  |  |  |  |
| **Sulfide** |  |  |  |  |  |  |  |  |  |  |
| Sulfide dehydrogenase *fccB* | 0 | 0 | 2 | 2 | 0 | 0 | 1 | 0 | 2 | 1 |
| Sulfide:quinone oxidoreductase *sqr* | 0 | 0 | 0 | 0 | 0 | 0 | 0 | 0 | 2 | 1 |
|  |  |  |  |  |  |  |  |  |  |  |
| **Sulfite** |  |  |  |  |  |  |  |  |  |  |
| ATP sulfurylase *sat* | 1 | 1 | 2 | 2 | 1 | 1 | 0 | 0 | 0 | 0 |
|  |  |  |  |  |  |  |  |  |  |  |
| **Accessory proteins** |  |  |  |  |  |  |  |  |  |  |
| Sulfurtransferase *tusA* | 2 | 2 | 4 | 3 | 1 | 1 | 0 | 0 | 0 | 0 |
| Sulfur transfer complex *TusD* | 0 | 0 | 1 | 1 | 0 | 0 | 0 | 0 | 13 | 0 |

^1^Abbreviations denote: Ac, *Acidithiobacillus caldus*-like; St, *Sulfobacillus thermosulfidooxidans*-like; Af, *Acidithiobacillus ferrivorans*-like; Cd, *Cuniculiplasma divulgatum*-like; and F, *Ferroplasma-*like.

**Supplemental File 8. Genes attributed energy conservation and potential strategies to electron transfer to the anode encoded in the MAGs^1^.**

|  |  | **MAGs identified in the metagenomes^2^** | | | | | | | | |
| --- | --- | --- | --- | --- | --- | --- | --- | --- | --- | --- |
| **Gene** | **S1_MAG1_Ac** | **S2_MAG1_Ac** | **S1_MAG2_St** | **S2_MAG2_St** | **S1_MAG3_Af** | **S2_MAG3_Af** | **S1_MAG4_Cd** | **S2_MAG4_Cd** | **S1_MAG5_F** | **S2_MAG5_F** |
| **NADH dehydrogenase** |  |  |  |  |  |  |  |  |  |  |
| NAD(P)H-quinone oxidoreductase *ndhC* | 2 | 2 | 1 | 1 | 2 | 2 | 1 | 1 | 0 | 1 |
| NAD(P)H-quinone oxidoreductase *ndhD* | 0 | 0 | 0 | 0 | 0 | 0 | 1 | 0 | 0 | 0 |
| NAD(P)H-quinone oxidoreductase *ndhE* | 0 | 0 | 0 | 0 | 0 | 0 | 0 | 0 | 0 | 1 |
| NAD(P)H-quinone oxidoreductase *ndhI* | 0 | 0 | 0 | 0 | 0 | 0 | 3 | 3 | 0 | 1 |
| NAD(P)H-quinone oxidoreductase *ndhK* | 0 | 0 | 0 | 0 | 0 | 0 | 0 | 0 | 0 | 1 |
| NADH-quinone oxidoreductase *nuoB* | 2 | 2 | 0 | 0 | 2 | 2 | 0 | 0 | 0 | 0 |
| NADH-quinone oxidoreductase *nuoC1* | 1 | 1 | 1 | 1 | 1 | 1 | 1 | 1 | 0 | 0 |
| NADH-quinone oxidoreductase *nuoC* | 1 | 1 | 0 | 0 | 1 | 1 | 0 | 0 | 0 | 1 |
| NADH-quinone oxidoreductase *nuoD* | 1 | 1 | 0 | 0 | 1 | 1 | 0 | 0 | 0 | 0 |
| NADH-quinone oxidoreductase *nuoCD* | 0 | 0 | 0 | 0 | 0 | 0 | 1 | 1 | 4 | 2 |
| NADH-quinone oxidoreductase *nuoE* | 1 | 1 | 1 | 1 | 1 | 1 | 0 | 0 | 0 | 0 |
| NADH-quinone oxidoreductase *nuoF* | 1 | 1 | 0 | 0 | 2 | 1 | 0 | 0 | 0 | 0 |
| NADH-quinone oxidoreductase *nuoG* | 1 | 1 | 0 | 0 | 1 | 1 | 0 | 0 | 0 | 0 |
| NADH-quinone oxidoreductase *nuoH* | 2 | 2 | 0 | 1 | 2 | 1 | 1 | 1 | 0 | 1 |
| NADH-quinone oxidoreductase *nuoI* | 2 | 2 | 0 | 0 | 2 | 2 | 0 | 0 | 0 | 0 |
| NADH-quinone oxidoreductase *nuoJ* | 2 | 1 | 1 | 1 | 1 | 2 | 2 | 2 | 0 | 1 |
| NADH-quinone oxidoreductase *nuoK* | 1 | 2 | 1 | 1 | 2 | 2 | 1 | 1 | 0 | 0 |
| NADH-quinone oxidoreductase *nuoL* | 5 | 5 | 0 | 0 | 4 | 5 | 1 | 1 | 0 | 1 |
| NADH-quinone oxidoreductase *nuoM* | 3 | 3 | 1 | 1 | 2 | 3 | 0 | 1 | 0 | 1 |
| NADH-quinone oxidoreductase *nuoN* | 2 | 2 | 1 | 1 | 2 | 2 | 1 | 1 | 0 | 1 |
| NADH-quinone oxidoreductase *nqo1* | 1 | 1 | 1 | 1 | 0 | 0 | 0 | 0 | 0 | 0 |
| NADH-quinone oxidoreductase *nqo2* | 1 | 1 | 0 | 0 | 1 | 1 | 0 | 0 | 0 | 0 |
| NADH-quinone oxidoreductase *nqo3* | 1 | 1 | 0 | 1 | 1 | 1 | 0 | 0 | 0 | 0 |
| NADH-quinone oxidoreductase *nqo4* | 0 | 0 | 1 | 1 | 0 | 0 | 1 | 1 | 0 | 1 |
| NADH-quinone oxidoreductase *nqo6* | 0 | 0 | 1 | 1 | 0 | 0 | 1 | 2 | 0 | 1 |
| NADH-quinone oxidoreductase *nqo8* | 0 | 0 | 1 | 0 | 0 | 0 | 0 | 0 | 0 | 0 |
| NADH-quinone oxidoreductase *nqo9* | 0 | 0 | 1 | 1 | 0 | 0 | 0 | 0 | 0 | 1 |
| NADH-quinone oxidoreductase *nqo12* | 0 | 0 | 2 | 1 | 0 | 0 | 0 | 0 | 0 | 0 |
| NADH:ubiquinone oxidoreductase J | 1 | 0 | 0 | 0 | 0 | 0 | 0 | 0 | 0 | 0 |
| NADH:ubiquinone oxidoreductase H | 0 | 0 | 0 | 0 | 0 | 0 | 0 | 0 | 4 | 0 |
|  |  |  |  |  |  |  |  |  |  |  |
| **Succinate dehydrogenase / fumarate reductase** | | | |  |  |  |  |  |  |  |
| Succinate dehydrogenase *sdhA* | 1 | 1 | 1 | 1 | 1 | 1 | 1 | 1 | 0 | 1 |
| Succinate dehydrogenase *sdhB* | 2 | 2 | 3 | 3 | 2 | 3 | 0 | 0 | 0 | 2 |
| Fumarate reductase *frdB* | 0 | 0 | 1 | 1 | 0 | 0 | 1 | 1 | 0 | 1 |
| Succinate dehydrogenase/fumarate reductase Fe-S | 0 | 0 | 0 | 0 | 0 | 0 | 0 | 0 | 1 | 0 |
|  |  |  |  |  |  |  |  |  |  |  |
| **Quinone** |  |  |  |  |  |  |  |  |  |  |
| 4-hydroxybenzoate octaprenyltransferase *ubiA* | 1 | 1 | 0 | 0 | 1 | 1 | 1 | 2 | 0 | 2 |
| Putative protein kinase *ubiB* | 0 | 1 | 3 | 3 | 1 | 1 | 1 | 1 | 3 | 1 |
| 3-octaprenyl-4-hydroxybenzoate carboxy-lyase *ubiD* | 1 | 1 | 0 | 0 | 1 | 1 | 1 | 1 | 2 | 1 |
| Ubiquinone biosynthesis O-methyltransferase *ubiG* | 2 | 2 | 0 | 0 | 1 | 1 | 0 | 0 | 0 | 0 |
| 2-octaprenyl-6-methoxyphenol hydroxylase *ubiH* | 1 | 1 | 0 | 0 | 1 | 1 | 0 | 0 | 0 | 0 |
| 2-octaprenylphenol hydroxylase *ubiI* | 1 | 1 | 0 | 0 | 1 | 1 | 0 | 0 | 0 | 0 |
| Quinol oxidase | 0 | 0 | 0 | 0 | 0 | 0 | 0 | 0 | 0 | 2 |
| Quinol oxidase subunit 2 precursor *qoxA* | 0 | 0 | 1 | 1 | 0 | 0 | 0 | 0 | 0 | 0 |
| Quinol oxidase subunit 1 *qoxB* | 0 | 0 | 1 | 1 | 0 | 0 | 0 | 1 | 0 | 0 |
| Quinol oxidase subunit 3 *qoxC* | 0 | 0 | 1 | 1 | 0 | 0 | 0 | 0 | 0 | 0 |
| Quinol oxidase subunit 4 *qoxD* | 0 | 0 | 1 | 1 | 0 | 0 | 0 | 0 | 0 | 0 |
|  |  |  |  |  |  |  |  |  |  |  |
| **Cytochrome *bo*_3_** |  |  |  |  |  |  |  |  |  |  |
| Cytochrome *o* ubiquinol oxidase IV | 2 | 2 | 0 | 0 | 2 | 2 | 0 | 0 | 0 | 0 |
| Cytochrome *bo*_3_ ubiquinol oxidase *cyoC* | 3 | 3 | 0 | 0 | 3 | 3 | 0 | 0 | 0 | 0 |
| Cytochrome *bo*_3_ ubiquinol oxidase *cyoB* | 3 | 3 | 0 | 0 | 3 | 3 | 0 | 0 | 0 | 0 |
| Cytochrome *bo*_3_ ubiquinol oxidase *cyoA* | 2 | 3 | 0 | 0 | 3 | 3 | 0 | 0 | 0 | 0 |
|  |  |  |  |  |  |  |  |  |  |  |
| **Cytochrome *bd*** |  |  |  |  |  |  |  |  |  |  |
| Cytochrome *bd*-II ubiquinol oxidase *appC* | 4 | 4 | 2 | 2 | 0 | 0 | 0 | 0 | 0 | 0 |
| Cytochrome *bd*-I ubiquinol oxidase *cydB* | 6 | 6 | 3 | 3 | 0 | 0 | 0 | 0 | 0 | 0 |
| Cytochrome *bd*-I ubiquinol oxidase *cydA* | 2 | 2 | 1 | 1 | 0 | 0 | 0 | 0 | 0 | 0 |
| ATP-binding/permease protein *cydD* | 0 | 1 | 0 | 0 | 0 | 0 | 0 | 0 | 0 | 0 |
| cytochrome bd menaquinol oxidase I *ythA* | 0 | 0 | 0 | 0 | 0 | 0 | 2 | 2 | 0 | 1 |
|  |  |  |  |  |  |  |  |  |  |  |
| **Cytochrome *bc*** |  |  |  |  |  |  |  |  |  |  |
| Menaquinol-cytochrome *c* reductase cytochrome *b* *qcrB* | 0 | 0 | 1 | 1 | 0 | 0 | 0 | 0 | 0 | 0 |
|  |  |  |  |  |  |  |  |  |  |  |
| **Prokaryotic cytochrome *b*_561_** |  |  |  |  |  |  |  |  |  |  |
| Prokaryotic cytochrome *b*_561_ | 0 | 0 | 0 | 0 | 4 | 4 | 0 | 0 | 0 | 0 |
|  |  |  |  |  |  |  |  |  |  |  |
| **Cytochrome *c*** |  |  |  |  |  |  |  |  |  |  |
| CcmB protein *ccmB* | 0 | 0 | 1 | 1 | 0 | 0 | 0 | 0 | 0 | 0 |
| Heme exporter protein C *ccmC* | 0 | 0 | 1 | 1 | 0 | 0 | 0 | 0 | 0 | 0 |
| Cytochrome *c* biogenesis protein *ccmE* | 0 | 0 | 1 | 1 | 0 | 0 | 0 | 0 | 0 | 0 |
| Cytochrome *c* biogenesis protein *ccmF* | 0 | 0 | 1 | 1 | 0 | 0 | 0 | 0 | 0 | 0 |
| Cytochrome *c* | 0 | 0 | 2 | 1 | 0 | 0 | 0 | 0 | 0 | 0 |
| Cytochrome *c*_1_ family | 0 | 0 | 0 | 0 | 1 | 1 | 0 | 0 | 0 | 0 |
| Cytochrome *c* assembly protein | 0 | 0 | 0 | 1 | 1 | 0 | 0 | 0 | 0 | 0 |
| Cytochrome *c*_552_ precursor *cyc1* | 1 | 1 | 0 | 0 | 2 | 2 | 0 | 0 | 0 | 0 |
| Ubiquinol-cytochrome *c* reductase iron-sulfur subunit *petA* | 0 | 0 | 0 | 0 | 1 | 1 | 0 | 0 | 0 | 0 |
| Cytochrome *b*_6_ *petB* | 1 | 1 | 1 | 1 |  | 0 | 1 | 1 | 0 | 1 |
| Cytochrome *b*_6_*f* *petC* | 0 | 0 | 0 | 0 | 0 | 0 | 0 | 0 | 0 | 2 |
| Cytochrome *c* biogenesis protein *ccsA* | 3 | 3 | 0 | 0 | 1 | 2 | 0 | 0 | 0 | 0 |
| Cytochrome *c* biogenesis protein *ccsB* | 1 | 2 | 0 | 0 | 1 | 1 | 0 | 0 | 0 | 0 |
|  |  |  |  |  |  |  |  |  |  |  |
| **Cytochrome b/*c_1_*** |  |  |  |  |  |  |  |  |  |  |
| Cytochrome *b*/*c_1_* | 0 | 0 | 0 | 0 | 1 | 0 | 0 | 0 | 0 | 0 |
|  |  |  |  |  |  |  |  |  |  |  |
| **Cytochrome *cbb*_3_ oxidase** |  |  |  |  |  |  |  |  |  |  |
| *Cbb*_3_-type cytochrome *c* oxidase *fixP* | 0 | 0 | 1 | 1 | 0 | 0 | 0 | 0 | 0 | 0 |
|  |  |  |  |  |  |  |  |  |  |  |
| **Cytochrome P450** |  |  |  |  |  |  |  |  |  |  |
| Cytochrome P450 | 0 | 0 | 0 | 0 | 0 | 0 | 1 | 1 | 9 | 2 |
|  |  |  |  |  |  |  |  |  |  |  |
| **Cytochrome *c* oxidase** |  |  |  |  |  |  |  |  |  |  |
| Heme A synthase *ctaA* | 0 | 0 | 2 | 2 | 0 | 0 | 0 | 0 | 0 | 0 |
| Protoheme IX farnesyltransferase *ctaB* | 0 | 0 | 1 | 1 | 0 | 2 | 1 | 1 | 0 | 1 |
| Cytochrome *c* oxidase subunit 2 *ctaC* | 0 | 0 | 3 | 3 | 0 | 0 | 0 | 0 | 0 | 0 |
| Cytochrome *c* oxidase subunit 1 *ctaD* | 0 | 0 | 2 | 2 | 0 | 0 | 0 | 0 | 0 | 0 |
| Cytochrome *c* oxidase *caa*_3_ assembly factor *ctaG* | 0 | 0 | 0 | 1 | 0 | 0 | 0 | 0 | 0 | 0 |
| Cytochrome *c* oxidase subunit III *coxIII* | 0 | 0 | 4 | 3 | 0 | 0 | 0 | 0 | 0 | 0 |
| Alternative cytochrome c oxidase *coxM* | 0 | 0 | 0 | 0 | 0 | 0 | 1 | 1 | 0 | 0 |
| Cytochrome *c* oxidase polypeptide I+III *caaA* | 0 | 0 | 0 | 1 | 0 | 0 | 1 | 0 | 0 | 0 |
| Cytochrome c oxidase polypeptide I | 0 | 0 | 0 | 0 | 0 | 0 |  |  | 1 |  |
| Cytochrome *c* oxidase polypeptide II | 0 | 0 | 0 | 0 | 0 | 0 | 1 | 1 | 0 | 0 |
|  |  |  |  |  |  |  |  |  |  |  |
| **ATP synthase** |  |  |  |  |  |  |  |  |  |  |
| ATP synthase subunit alpha *atpA* | 1 | 1 | 1 | 1 | 1 | 1 | 1 | 1 | 0 | 1 |
| ATP synthase subunit a *atpB* | 1 | 1 | 1 | 1 | 1 | 1 | 1 | 1 | 0 | 1 |
| ATP synthase epsilon chain *atpC* | 3 | 3 | 1 | 1 | 1 | 1 | 1 | 1 | 0 | 1 |
| ATP synthase subunit beta *atpD* | 1 | 1 | 1 | 1 | 1 | 1 | 1 | 1 | 0 | 1 |
| ATP synthase subunit c *atpE* | 1 | 1 | 1 | 1 | 1 | 1 | 1 | 1 | 0 | 1 |
| ATP synthase subunit b *atpF* | 1 | 1 | 1 | 1 | 1 | 1 | 1 | 1 | 0 | 0 |
| ATP synthase gamma chain *atpG* | 1 | 1 | 1 | 1 | 1 | 1 | 0 | 0 | 0 | 0 |
| ATP synthase subunit delta *atpH* | 1 | 1 | 1 | 1 | 1 | 1 | 0 | 0 | 0 | 0 |
| Putative F_0_F_1_-ATPase subunit (ATPase_gene1) | 0 | 0 | 1 | 1 | 0 | 0 | 0 | 0 | 0 | 0 |
| V-type ATP synthase subunit I *atpI* | 0 | 0 | 0 | 0 | 0 | 0 | 1 | 1 | 0 | 0 |
| V-type Na-ATPase subunit D *ntpD* | 0 | 0 | 0 | 0 | 0 | 0 | 1 | 1 | 0 | 0 |
| V-type Na-ATPase subunit B *ntpB* | 0 | 0 | 0 | 0 | 0 | 0 | 1 | 1 | 0 | 1 |
| V-type Na-ATPase subunit A *ntpA* | 0 | 0 | 0 | 0 | 0 | 0 | 1 | 1 | 0 | 1 |
| V-type ATP synthase subunit F *atpF* | 0 | 0 | 0 | 0 | 0 | 0 | 1 | 1 | 0 | 0 |
| V-type ATP synthase subunit C *atpC* | 0 | 0 | 0 | 0 | 0 | 0 | 1 | 1 | 2 | 1 |
| V-type ATP synthase subunit D | 0 | 0 | 0 | 0 | 0 | 0 | 0 | 0 | 2 | 1 |
| V-type ATP synthase subunit K *atpK* | 0 | 0 | 0 | 0 | 0 | 0 | 1 | 1 | 2 | 1 |
| V-type ATP synthase subunit E *atpE* | 0 | 0 | 0 | 0 | 0 | 0 | 1 | 1 | 2 | 1 |
| V-type ATP synthase subunit H | 0 | 0 | 0 | 0 | 0 | 0 | 0 | 0 | 2 | 1 |
| V-type ATP synthase subunit I | 0 | 0 | 0 | 0 | 0 | 0 | 1 | 1 | 2 | 1 |
| V-type ATP synthase subunit J | 0 | 0 | 0 | 0 | 0 | 0 | 1 | 1 |  |  |
| V-type ATP synthase alpha chain | 0 | 0 | 0 | 0 | 0 | 0 | 0 | 0 | 2 | 0 |
| V-type ATP synthase beta chain | 0 | 0 | 0 | 0 | 0 | 0 | 0 | 0 | 2 | 0 |
|  |  |  |  |  |  |  |  |  |  |  |
| **Pili formation** |  |  |  |  |  |  |  |  |  |  |
| Type 4 prepilin-like proteins leader peptide-processing enzyme *outO* | 1 | 2 | 0 | 0 | 1 | 1 | 0 | 0 | 0 | 0 |
| Type IV pilus biogenesis | 1 | 1 | 0 | 0 | 0 | 0 | 0 | 0 | 0 | 0 |
| Fimbrial protein precursor *pilE* | 1 | 1 | 0 | 0 | 1 | 1 | 0 | 0 | 0 | 0 |
| Twitching mobility protein *pilT* | 1 | 3 | 0 | 0 | 2 | 2 | 0 | 0 | 0 | 0 |
| Type IV pilus biogenesis and competence protein PilQ precursor pilQ | 1 | 1 | 0 | 0 | 1 | 1 | 0 | 0 | 0 | 0 |
| Pilus assembly protein PilO | 1 | 2 | 0 | 0 | 1 | 1 | 0 | 0 | 0 | 0 |
| Pilus assembly protein PilP | 1 | 1 | 0 | 0 | 1 | 1 | 0 | 0 | 0 | 0 |
| Flp/Fap pilin component | 0 | 0 | 1 | 1 | 0 | 0 | 0 | 0 | 0 | 0 |
|  |  |  |  |  |  |  |  |  |  |  |
|  | | |  |  |  |  |  |  |  |  |
| **Riboflavin/Flavin mononucleotide (FMN)** | | |  |  |  |  |  |  |  |  |
| GTP cyclohydrolase-2 *ribA* | 0 | 0 | 0 | 0 | 0 | 0 | 1 | 1 | 0 | 0 |
| 3-2C4-dihydroxy-2-butanone 4-phosphate synthase *ribB* | 0 | 0 | 0 | 0 | 0 | 0 | 0 | 0 | 1 | 1 |
| Riboflavin synthase *ribC* | 0 | 0 | 0 | 0 | 0 | 0 | 1 | 1 | 1 | 1 |
| Riboflavin biosynthesis protein *ribBA* | 1 | 1 | 1 | 1 | 1 | 1 | 2 | 2 | 0 | 1 |
| Riboflavin biosynthesis protein *ribD* | 1 | 1 | 1 | 1 | 1 | 1 | 1 | 1 | 0 | 1 |
| Riboflavin synthase *ribE* | 1 | 1 | 1 | 1 | 1 | 1 | 1 | 1 | 0 |  |
| Riboflavin biosynthesis protein *ribF* | 1 | 1 | 1 | 1 | 1 | 1 | 0 | 0 | 0 | 0 |
| Riboflavin biosynthesis protein *ribH* | 1 | 1 | 1 | 1 | 1 | 1 | 1 | 1 | 1 | 1 |
| Riboflavin kinase *ribK* | 0 | 0 | 0 | 0 | 0 | 0 | 1 | 1 | 2 | 0 |
| Energy-coupling factor transporter transmembrane protein *EcfT* | 0 | 0 | 1 | 1 | 0 | 0 | 1 | 0 | 0 | 0 |
|  |  |  |  |  |  |  |  |  |  |  |
| **Other redox active compounds** |  |  |  |  |  |  |  |  |  |  |
| O-succinylbenzoate synthase *menC* | 0 | 0 | 1 | 1 | 0 | 0 | 1 | 1 | 0 | 1 |
| 2-succinylbenzoate--CoA ligase *memE* | 0 | 0 | 0 | 1 | 0 | 0 | 0 | 0 | 0 | 1 |
| S-adenosyl-L-methione--menaquinol methyltransferase *memG/ubiE* | 4 | 4 | 7 | 6 | 3 | 3 | 4 | 4 | 0 | 3 |
| Flavoprotein-ubiquinone oxidoreductase | 0 | 0 | 0 | 0 | 1 | 0 | 1 | 1 | 0 | 1 |
| Electron transfer flavoprotein subunit beta *etfB* | 0 | 0 | 1 | 2 | 0 | 0 | 1 | 1 | 0 | 0 |
| Electron transfer flavoprotein subunit *YdiR* | 0 | 0 | 0 | 0 | 0 | 0 | 0 | 0 | 3 | 0 |
|  |  |  |  |  |  |  |  |  |  |  |
| **Phenazine biosynthesis** |  |  |  |  |  |  |  |  |  |  |
| Trans-2,3-dihydro-3-hydroxyanthranilate isomerase *phzF* | 0 | 0 | 1 | 1 | 0 | 0 | 0 | 0 | 0 | 0 |
|  |  |  |  |  |  |  |  |  |  |  |
| **Anaerobic polysulfide reduction** |  |  |  |  |  |  |  |  |  |  |
| Tetrathionate reductase *ttrA* | 0 | 0 | 0 | 0 | 0 | 1 | 0 | 0 | 0 | 0 |
| Tetrathionate reductase *ttrB* | 2 | 2 | 2 | 2 | 1 | 1 | 0 | 0 | 0 | 0 |
| Sulfite reductase *asrB* | 0 | 0 | 0 | 0 | 0 | 0 | 1 | 1 | 0 | 1 |
| Polysulfide reductase A *psrA* | 0 | 0 | 1 | 0 | 0 | 0 | 0 | 0 | 0 | 0 |
| Polysulphide reductase 2C *nrfD* | 0 | 0 | 2 | 2 | 0 | 0 | 0 | 0 | 0 | 0 |
|  |  |  |  |  |  |  |  |  |  |  |
| **Type II secretion system** |  |  |  |  |  |  |  |  |  |  |
| Type II secretion system protein E *epsE* | 2 | 2 | 0 | 0 | 1 | 2 | 0 | 0 | 0 | 0 |
| Type II secretion system protein E *epsF* | 1 | 1 | 2 | 2 | 1 | 2 | 0 | 0 | 0 | 0 |
| Type II secretion system protein D *outD/pulD* | 2 | 1 | 0 | 0 | 2 | 3 | 0 | 0 | 0 | 0 |

^1^The following systems were not present on any of the MAGs: the two porin cytochrome c systems involved in electron transfer by Geobacter sulfrureducens and Shewanella oneidensis; the phenazine based system in Pseudomonas spp.; and the pyrocyanin system utilized by Pseudomonas aeruginosa.

^2^Abbreviations denote: Ac, *Acidithiobacillus caldus*-like; St, *Sulfobacillus thermosulfidooxidans*-like; Af, *Acidithiobacillus ferrivorans*-like; Cd, *Cuniculiplasma divulgatum*-

like; and F, *Ferroplasma-*like.

**Supplemental File 9. Genes attributed to carbon fixation within the MAGs^1^.**

|  |  | **MAGs identified in the metagenomes^2^** | | | | | | | | | | | |
| --- | --- | --- | --- | --- | --- | --- | --- | --- | --- | --- | --- | --- | --- |
| **Gene** | **S1_MAG1_Ac** | | **S2_MAG1_Ac** | **S1_MAG2_St** | **S2_MAG2_St** | **S1_MAG3_Af** | **S2_MAG3_Af** | | **S1_MAG4_Cd** | **S2_MAG4_Cd** | **S1_MAG5_F** | | **S2_MAG5_F** |
| **Calvin-Benson-Bassham (CBB) cycle** | | | | | |  | |  | |  | |  | |
| RuBisCo *cbbM* | 1 | | 1 | 0 | 0 | 1 | 1 | | 0 | 0 | 0 | | 0 |
| RuBisCo *cbbS* | 1 | | 1 | 0 | 0 | 3 | 3 | | 0 | 0 | 0 | | 0 |
| RuBisCo *cbbL* | 1 | | 1 | 1 | 1 | 3 | 3 | | 0 | 0 | 0 | | 0 |
| RuBisCo small chain | 0 | | 0 | 1 | 1 | 0 | 0 | | 0 | 0 | 0 | | 0 |
| Major carboxysome shell protein *csoS1C* | 3 | | 1 | 0 | 0 | 0 | 1 | | 0 | 0 | 0 | | 0 |
| Major carboxysome shell protein *csoS1A* | 2 | | 2 | 0 | 0 | 6 | 4 | | 0 | 0 | 0 | | 0 |
| Carboxysome shell peptide mid-region | 1 | | 1 | 0 | 0 | 1 | 2 | | 0 | 0 | 0 | | 0 |
| Carboxysome shell carbonic anhydrase | 1 | | 1 | 0 | 0 | 1 | 2 | | 0 | 0 | 0 | | 0 |

^1^Other carbon dioxide fixation systems such as the reverse Krebs cycle, Wood–Ljungdahl pathway, and 3-Hydroxypropionate pathway were not identified in any of the MAGs.

^2^Abbreviations denote: Ac, *Acidithiobacillus caldus*-like; St, *Sulfobacillus thermosulfidooxidans*-like; Af, *Acidithiobacillus ferrivorans*-like; Cd, *Cuniculiplasma divulgatum*-

like; and F, *Ferroplasma-*like.

**Supplemental File 10. Genes attributed to adaptation and growth at low pH as well as metal resistance identified in the MAGs.**

|  |  | **MAGs identified in the metagenomes^1^** | | | | | | | | |
| --- | --- | --- | --- | --- | --- | --- | --- | --- | --- | --- |
| **Gene** | **S1_MAG1_Ac** | **S2_MAG1_Ac** | **S1_MAG2_St** | **S2_MAG2_St** | **S1_MAG3_Af** | **S2_MAG3_Af** | **S1_MAG4_Cd** | **S2_MAG4_Cd** | **S2_MAG5_F** | **S2_MAG5_F** |
| **Role of potassium in the internal positive membrane potential** | | | | | | | | | | |
| K^+^-transporting ATPase C chain | 2 | 2 | 2 | 2 | 1 | 1 | 1 | 1 | 0 | 1 |
| K^+^-transporting ATPase B chain | 2 | 2 | 2 | 2 | 1 | 1 | 1 | 1 | 0 | 1 |
| K^+^-transporting ATPase A chain | 3 | 3 | 2 | 2 | 1 | 1 | 1 | 1 | 0 | 1 |
| Sensor protein gene *kdpD* | 1 | 1 | 1 | 1 | 1 | 2 | 0 | 0 | 0 | 0 |
| Regulatory protein *kdpE* | 2 | 2 | 1 | 1 | 1 | 2 | 0 | 0 | 0 | 0 |
| K^+^-transporter membrane component | 1 | 1 | 0 | 0 | 0 | 0 | 0 | 0 | 0 |  |
| Low affinity K^+^ transport *kup* | 1 | 1 | 0 | 0 | 0 | 0 | 0 | 0 | 0 | 0 |
| Voltage-gated K^+^ channel *kch* | 2 | 2 | 0 | 0 | 2 | 2 | 1 | 1 | 0 | 1 |
| pH-gated potassium channel *kcsA* | 0 | 0 | 0 | 0 | 0 | 0 | 0 | 0 | 0 | 1 |
| Calcium-gated K^+^ channel *MthK* | 0 | 0 | 0 | 0 | 0 | 0 | 0 | 0 | 5 | 0 |
|  |  |  |  |  |  |  |  |  |  |  |
| **Proton transporters** |  |  |  |  |  |  |  |  |  |  |
| Na^+^/H^+^ antiporter *mrpD* | 0 | 0 | 1 | 1 | 0 | 0 | 0 | 0 | 0 | 0 |
| Na^+^/H^+^ antiporter *nhaA* | 0 | 0 | 0 | 0 | 1 | 1 | 0 | 0 | 0 | 0 |
| Na^+^/H^+^ antiporter *nhaD* | 0 | 0 | 1 | 1 | 0 | 0 | 0 | 0 | 0 | 0 |
| Na^+^/H^+^ antiporter *nhaG* | 1 | 1 | 0 | 0 | 2 | 2 | 0 | 0 | 0 | 0 |
| Na^+^/H^+^ antiporter 1 | 0 | 0 | 0 | 0 | 0 | 0 | 1 | 1 | 2 | 1 |
| K^+^/H^+^ antiporter *nhaP* | 0 | 0 | 0 | 0 | 0 | 0 | 1 | 1 | 0 | 1 |
| K^+^/H^+^ antiporter | 0 | 0 | 1 | 1 | 0 | 0 | 0 | 0 | 0 | 0 |
| High-affinity Na^+^/H^+^ antiporter *nhaS* | 0 | 0 | 0 | 0 | 1 | 1 | 0 | 0 | 0 | 0 |
| H^+^/Cl^-^ exchange transporter *clcA* | 2 | 2 | 1 | 2 | 3 | 3 | 1 | 1 | 0 | 0 |
|  |  |  |  |  |  |  |  |  |  |  |
| **Proton consuming reactions** |  |  |  |  |  |  |  |  |  |  |
| Glutamate decarboxylase *gadA* | 0 | 0 | 1 | 1 | 0 | 0 | 0 | 0 | 0 | 0 |
| Glutamate decarboxylase *gadB* | 1 | 1 | 0 | 0 | 1 | 1 | 1 | 1 | 0 | 2 |
| Glutamate decarboxylase *gadC* | 0 | 0 | 1 | 1 | 0 | 0 | 0 | 0 | 0 | 0 |
| GABA permease *gabP* | 0 | 0 | 2 | 2 | 0 | 0 | 0 | 0 | 0 | 1 |
|  |  |  |  |  |  |  |  |  | 0 |  |
| **Spermidine** |  |  |  |  |  |  |  |  | 0 |  |
| Carboxynorspermidine synthase | 0 | 0 | 1 | 1 | 0 | 0 | 0 | 0 | 0 | 0 |
| Spermidine/putrescine-binding *potD* | 0 | 0 | 1 | 1 | 0 | 0 | 0 | 0 | 0 | 0 |
| Spermidine/putrescine-binding *potB* | 0 | 0 | 1 | 1 | 0 | 0 | 0 | 0 | 0 | 0 |
| Spermidine/putrescine-binding *potA* | 0 | 0 | 3 | 3 | 1 | 1 | 0 | 0 | 0 | 0 |
| Spermidine synthase *speE* | 1 | 1 | 2 | 2 | 2 | 1 | 0 | 1 | 0 | 1 |
| Spermidine N_1_-acetyltransferase *speG* | 0 | 0 | 0 | 0 | 0 | 0 | 1 | 1 | 0 | 0 |
| S-adenosylmethionine decarboxylase *speH* | 0 | 0 | 0 | 0 | 1 | 0 | 0 | 0 | 3 | 0 |
|  |  |  |  |  |  |  |  |  |  |  |
| **Acid shock/resistance** |  |  |  |  |  |  |  |  |  |  |
| Acid-resistance membrane protein | 1 | 1 | 0 | 0 | 1 | 1 | 0 | 0 | 0 | 0 |
| Acid shock protein *asr* | 1 | 1 | 0 | 0 | 0 | 0 | 0 | 0 | 0 | 0 |
|  |  |  |  |  |  |  |  |  |  |  |
| **Arsenic resistance** |  |  |  |  |  |  |  |  |  |  |
| Transcriptional repressor *arsR* | 1 | 1 | 1 | 1 | 0 | 0 | 2 | 2 | 8 | 1 |
| As efflux membrane subunit *arsB* | 1 | 1 | 1 | 1 | 3 | 3 | 1 | 1 | 0 | 1 |
| Arsenate reductase *arsC* | 0 | 0 | 1 | 1 | 1 | 1 | 1 | 1 | 0 | 0 |
| ATPase *arsA* | 0 | 0 | 2 | 2 | 1 | 1 | 1 | 1 | 0 | 1 |
| Chaperone *arsD* | 0 | 0 | 1 | 1 | 1 | 1 | 0 | 0 | 0 | 0 |
| Arsenite methyltransferase | 0 | 0 | 0 | 1 | 0 | 0 | 0 | 0 | 0 | 0 |
|  |  |  |  |  |  |  |  |  |  |  |
| **Copper resistance** |  |  |  |  |  |  |  |  |  |  |
| Cu(I) transcriptional repressor *csoR* | 2 | 2 | 2 | 2 | 0 | 0 | 0 | 0 | 0 | 0 |
| Cu-exporting P-type ATPase *copA* | 1 | 1 | 2 | 2 | 1 | 1 | 2 | 2 | 5 | 2 |
| Periplasmic Cu-binding *copZ* | 1 | 1 | 1 | 1 | 0 | 0 | 0 | 0 | 0 | 0 |
| Cu-exporting P-type ATPase *copB* | 1 | 1 | 0 | 0 | 0 | 0 | 2 | 2 | 8 | 2 |
| Cu-exporting P-type ATPase *actP* | 2 | 2 | 0 | 0 | 1 | 1 | 0 | 0 | 0 | 0 |
|  |  |  |  |  |  |  |  |  |  |  |
| **Copper/silver resistance** |  |  |  |  |  |  |  |  |  |  |
| Sensor kinase *cusS* | 0 | 0 | 0 | 0 | 0 | 1 | 0 | 0 | 0 | 0 |
| Membrane fusion protein *cusB* | 3 | 2 | 0 | 0 | 1 | 2 | 0 | 0 | 0 | 0 |
| RND protein superfamily *cusA* | 2 | 1 | 0 | 0 | 1 | 2 | 0 | 0 | 0 | 0 |
|  |  |  |  |  |  |  |  |  |  |  |
| **Silver** |  |  |  |  |  |  |  |  |  |  |
| Ag exporting P-type ATPase *silP* | 0 | 0 | 0 | 0 | 0 | 0 | 1 | 1 | 0 | 0 |
|  |  |  |  |  |  |  |  |  |  |  |
| **Cadmium** |  |  |  |  |  |  |  |  |  |  |
| Cd resistance regulator *cadC* | 1 | 0 | 0 | 0 | 0 | 0 | 0 | 0 | 0 | 0 |
| Cadmium-induced *cadI* | 1 | 1 | 0 | 0 | 0 | 0 | 0 | 0 | 0 | 0 |
|  |  |  |  |  |  |  |  |  |  |  |
| **Cadmium/cobalt/zinc resistance** | | |  |  |  |  |  |  |  |  |
| Sensor protein *czcS* | 0 | 0 | 0 | 0 | 1 | 0 | 0 | 0 | 0 | 0 |
| Transcriptional activator *czcR* | 0 | 0 | 0 | 0 | 1 | 1 | 0 | 0 | 0 | 0 |
| Cd/Co/Zn H^+^-K^+^ efflux *czcA* | 4 | 4 | 0 | 0 | 0 | 0 | 0 | 0 | 0 | 0 |
| Cd/Co/Zn precursor *czcC* | 2 | 1 | 0 | 0 | 1 | 2 | 0 | 0 | 0 | 0 |
| Cd/Co/Zn H^+^-K^+^ antiporter *czcD* | 3 | 3 | 1 | 1 | 0 | 1 | 1 | 1 | 0 | 1 |
|  |  |  |  |  |  |  |  |  |  |  |
| **Nickel/cobalt resistance** |  |  |  |  |  |  |  |  |  |  |
| Ni/Co resistance protein *cnrB* | 2 | 2 | 0 | 0 | 1 | 1 | 0 | 0 | 0 | 0 |
| Ni/Co efflux protein *rcnA* | 1 | 1 | 0 | 0 | 0 | 0 | 0 | 0 | 0 | 0 |
|  |  |  |  |  |  |  |  |  |  |  |
| **Magnesium/cobalt resistance** | | |  |  |  |  |  |  |  |  |
| Mg/Co efflux protein *corC* | 2 | 2 | 1 | 1 | 2 | 2 | 0 | 0 | 0 | 0 |
| Transcriptional regulator *mntR* | 0 | 0 | 1 | 1 | 0 | 0 | 0 | 1 | 5 | 1 |
| Divalent metal transporter *mntH* | 1 | 1 | 4 | 5 | 1 | 2 | 1 | 1 | 3 | 1 |
| Manganese efflux pump *mntP* | 0 | 0 | 1 | 1 | 0 | 0 | 0 | 0 | 0 | 0 |
|  |  |  |  |  |  |  |  |  |  |  |
| **Mercury resistance** |  |  |  |  |  |  |  |  |  |  |
| Regulator *merR* | 4 | 4 | 1 | 1 | 3 | 3 | 0 | 0 | 0 | 0 |
| Reductase *merA* | 2 | 1 | 1 | 1 | 1 | 1 | 2 | 1 | 0 | 2 |
| Resistance protein *merC* | 1 | 2 | 0 | 0 | 0 | 0 | 0 | 0 | 0 | 1 |
| Transport protein periplasmic component precursor *merP* | 1 | 1 | 0 | 0 | 1 | 1 | 0 | 0 | 0 | 0 |
| Transport protein *merT* | 1 | 1 | 0 | 0 | 1 | 1 | 0 | 0 | 0 | 0 |
|  |  |  |  |  |  |  |  |  |  |  |
| **Chromate resistance** |  |  |  |  |  |  |  |  |  |  |
| Chromate resistance exporter | 0 | 0 | 1 | 1 | 0 | 0 | 1 | 1 | 0 | 0 |
| Chromate transport protein *chrA* | 0 | 0 | 2 | 2 | 0 | 0 | 0 | 0 | 0 | 0 |
|  |  |  |  |  |  |  |  |  |  |  |
| **Cation tolerance** |  |  |  |  |  |  |  |  |  |  |
| Divalent-cation tolerance *cutA* | 1 | 1 | 0 | 0 | 0 | 0 | 0 | 0 | 4 | 2 |

^1^Abbreviations denote: Ac, *Acidithiobacillus caldus*-like; St, *Sulfobacillus thermosulfidooxidans*-like; Af, *Acidithiobacillus ferrivorans*-like; Cd, *Cuniculiplasma divulgatum*-like; and F, *Ferroplasma-*like.
